# DNA methylation and stroke prognosis: an epigenome-wide association study

**DOI:** 10.1186/s13148-024-01690-2

**Published:** 2024-06-06

**Authors:** Joan Jiménez-Balado, Isabel Fernández-Pérez, Cristina Gallego-Fábrega, Uxue Lazcano, Carolina Soriano-Tárraga, Marta Vallverdú-Prats, Marina Mola-Caminal, Lucía Rey-Álvarez, Adrià Macias-Gómez, Antoni Suárez-Pérez, Eva Giralt-Steinhauer, Ana Rodríguez-Campello, Elisa Cuadrado-Godia, Ángel Ois, Manel Esteller, Jaume Roquer, Israel Fernández-Cadenas, Jordi Jiménez-Conde

**Affiliations:** 1 Neurovascular Research Group, Department of Neurology, Hospital del Mar Research Institute, C/ del Dr. Aiguader, 88, 08003 Barcelona, Spain; 2 Institut d’Investigació Biomèdica Sant Pau (IIB SANT PAU), Sant Quintí, Barcelona, Spain; 3 Center for Cooperative Research in Biosciences (CIC bioGUNE), Basque Research and Technology Alliance (BRTA), Biscaia, Spain; 4 Department of Psychiatry, NeuroGenomics and Informatics, Washington University School of Medicine, St. Louis, MO 63110 USA; 5 Unit of Medical Epidemiology, Department of Surgical Sciences, Uppsala University, Uppsala, Sweden; 6 Medicine Department, DCEXS-Universitat Pompeu Fabra (UPF), 08002 Barcelona, Spain; 7 Cancer Epigenetics Group, Josep Carreras Leukaemia Research Institute (IJC), Badalona, Barcelona, Catalonia Spain; 8 Centro de Investigacion Biomedica en Red Cancer (CIBERONC), Madrid, Spain; 9 Institucio Catalana de Recerca I Estudis Avançats (ICREA), Barcelona, Catalonia Spain; 10 Physiological Sciences Department, School of Medicine and Health Sciences, University of Barcelona (UB), Barcelona, Catalonia Spain; 11 Medicine Department, Autonomous University of Barcelona, Barcelona, Spain

**Keywords:** Epigenetics, Stroke outcome, Thrombospondin-2, DNA methylation

## Abstract

**Background and aims:**

Stroke is the leading cause of adult-onset disability. Although clinical factors influence stroke outcome, there is a significant variability among individuals that may be attributed to genetics and epigenetics, including DNA methylation (DNAm). We aimed to study the association between DNAm and stroke prognosis.

**Methods and results:**

To that aim, we conducted a two-phase study (discovery-replication and meta-analysis) in Caucasian patients with ischemic stroke from two independent centers (BasicMar [discovery, *N* = 316] and St. Pau [replication, *N* = 92]). Functional outcome was assessed using the modified Rankin Scale (mRS) at three months after stroke, being poor outcome defined as mRS > 2. DNAm was determined using the 450K and EPIC BeadChips in whole-blood samples collected within the first 24 h. We searched for differentially methylated positions (DMPs) in 370,344 CpGs, and candidates below *p*-value < 10^–5^ were subsequently tested in the replication cohort. We then meta-analyzed DMP results from both cohorts and used them to identify differentially methylated regions (DMRs).

After doing the epigenome-wide association study, we found 29 DMPs at *p*-value < 10^–5^ and one of them was replicated: cg24391982, annotated to thrombospondin-2 (*THBS2*) gene (*p*-value_discovery_ = 1.54·10^–6^; *p*-value_replication_ = 9.17·10^–4^; *p*-value_meta-analysis_ = 6.39·10^–9^). Besides, four DMRs were identified in patients with poor outcome annotated to zinc finger protein 57 homolog (*ZFP57*), Arachidonate 12-Lipoxygenase 12S Type (*ALOX12*), ABI Family Member 3 (*ABI3*) and Allantoicase (*ALLC*) genes (*p*-value < 1·10^–9^ in all cases).

**Discussion:**

Patients with poor outcome showed a DMP at *THBS2* and four DMRs annotated to *ZFP57, ALOX12, ABI3* and *ALLC* genes. This suggests an association between stroke outcome and DNAm, which may help identify new stroke recovery mechanisms.

**Supplementary Information:**

The online version contains supplementary material available at 10.1186/s13148-024-01690-2.

## Introduction and background

Ischemic stroke (IS) is the leading cause of adult-onset disability and the second cause of death according to current epidemiological data [1]. Specifically, one out of four individuals will experience a stroke during their life, and one-third of stroke survivors will have some degree of long-term disability [2]. Although stroke incidence has decreased during the last few decades thanks to the improvement in secondary prevention strategies, its prevalence is higher due to the increase in life expectancy [3], causing significant direct and indirect economic costs for the healthcare system and reducing the quality of life of patients and caregivers [4].

After the acute phase, there is a huge variation in stroke outcome among patients. A significant proportion of this variation can be explained considering clinical variables such as history of vascular risk factors, clinical management, previous cerebrovascular burden, among others [5]. However, even after considering these factors, there is still a considerable degree of variability between individuals with similar clinical conditions, which is thought to be caused by genetics [6]. Previous research indeed found 89 loci associated with stroke risk [7]. Role of genetics in stroke recovery, on the other hand, has been less studied, and current knowledge is mostly restricted to two genome-wide association studies (GWAS) [8, 9], which provided insight on the biological mechanisms involved in stroke outcome and proposed potential therapeutical targets.

Moreover, during the last decade there has been a growing interest in stroke epigenetics, which are hereditable and modifiable factors that regulate gene expression without altering DNA sequence [10]. DNA methylation (DNAm), as the addition of a methyl group in a cytosine-phosphate-guanine context (CpG), is related to gene silencing or expression, and it represents the most studied epigenetic mechanism in stroke research. A recent article indeed confirmed specific DNAm signatures in patients with stroke as compared to individuals without [11]. However, epigenetics of stroke recovery has been rarely studied, and only one previous project interrogated the role of DNAm in early neurological evolution, as the difference between initial and at discharge National Institute of Health Stroke Scale (NIHSS). This study found that differences in the methylation levels in the *EXOC4* gene yielded a worse neurological course after stroke [12]. On the other hand, to our better knowledge, no study has reported a relationship between DNAm and 3-month outcome, as measured by the modified Rankin Scale (mRS), which might provide important insight into the epigenetic regulation of stroke recovery after the acute phase. Therefore, our main objective was to analyze the relation between DNAm and stroke outcome to achieve a better understanding on the role of epigenetics in stroke recovery.

## Methods

**Fig. 1:**
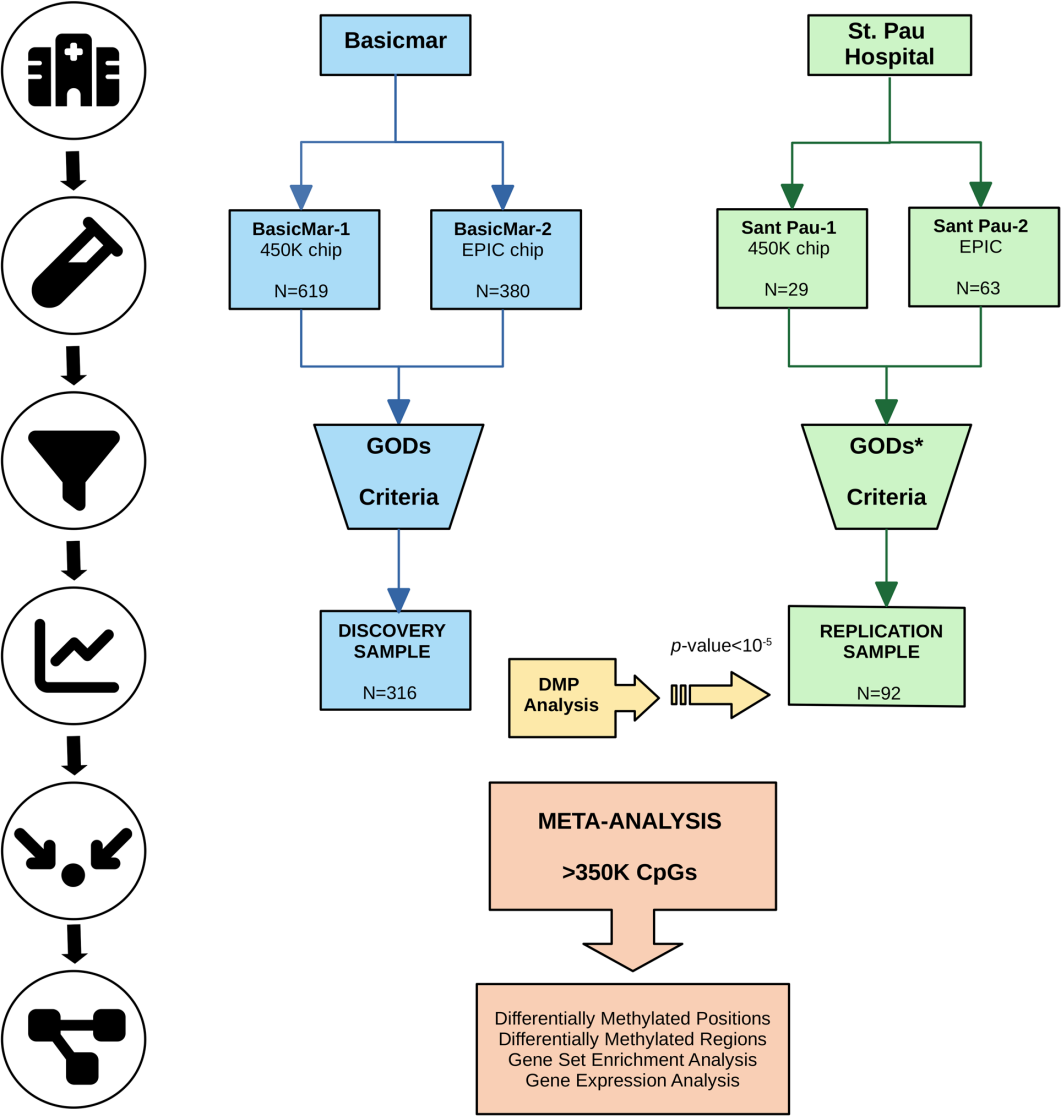
Study design diagram. Discovery sample was conformed by two cohorts of patients with stroke, BasicMar-1 (450K Illumina Chip) and BasicMar-2 (EPIC Illumina Chip). Replication phase consisted in two cohorts as well (St. Pau-1, 450 K Illumina Chip; and St. Pau-2, EPIC Illumina Chip). In both cases we filtered cases based on strict stringent criteria defined in a previous study (supplemental methods). We then searched Differentially Methylated Positions (DMPs) in the discovery cohort, and those CpG candidates having a *p*-value < 10^–5^ were tested for significance in the replication phase. Both stages were meta-analyzed to search for differentially methylated regions (DMRs) and biological pathways. Gene expression analyses were ran for the replicated DMPs. *In the replication phase, patients could not be rule out according to previous functional status and stroke location, as this cohort did not collect these data

A two-phase EWAS was conducted in stroke patients to identify differentially methylated positions (DMPs) associated with stroke outcome. The study consisted of a discovery-replication phases followed by a meta-analysis. Subsequently, we explored the differentially methylated regions (DMRs) and the enriched biological pathways. We show a visual representation of the study design in Fig. 1. Full methods are available in supplemental material.

### Setting and participants

#### Discovery sample

European-ancestry IS patients were selected from 2 cohorts nested in the BasicMar register (BasicMar-1 [*N* = 619] & BasicMar-2 [*N* = 380]) following strict inclusion criteria [9]: (1) acute anterior ischemic stroke patients; (2) modified Rankin Scale (mRS) < 3; (3) initial NIHSS > 2. The exclusion criteria were: (1) lacunar stroke etiology and (2) recurrence of stroke during the follow-up period. For more details consult supplementary methods. A total of 323 subjects met these inclusion and exclusion criteria (Fig. 1, discovery sample).

#### Replication sample

Replication sample consisted in a subset of 92 patients who belong to the St. Pau cohort (St. Pau-1 [*N* = 29] & St. Pau-2 [*N* = 63]). Selection criteria were the same as discovery sample except for the following data, which was not available in the replication cohort: (1) previous functional status; (2) whether the infarct was posterior or anterior. Therefore, patients were not rule-out based on these criteria.

### Clinical severity and functional outcome

Stroke severity and functional evaluation were assessed by stroke neurologists. Clinical severity was evaluated using the NIHSS [13] upon arrival at the hospital, at 24 h and at 3 months after stroke onset. Previous stroke functional outcome was scored according to the mRS [14]. The primary endpoint was the functional outcome after 3 months. Poor outcome was defined as a mRS score from 3 to 6. Etiological stroke subtypes were classified according to TOAST criteria [15].

### DNA methylation quantification

DNA was extracted from whole peripheral blood collected in 10-mL EDTA tubes during the first 24 h after stroke onset. For the discovery study, BasicMar-1 (*N* = 252), DNAm was analyzed using the Human Methylation 450 K Beadchip (Illumina, Netherlands, Eindhoven; more than 450,000 CpGs) in two different technical runs, while for BasicMar-2 (*N* = 71) we used the Infinium Methylation EPIC Beadchip (Illumina, Netherlands, Eindhoven; more than 850,000 CpGs) in one technical run. Therefore, a total of 323 subjects were included in the discovery phase before quality controls (Fig. 1). For the replication study, we used the 450 K Beadchip for St. Pau-1 (*N* = 29) and the EPIC Beadchip for St. Pau-2 (*N* = 63).

### DNA methylation quality controls

Intensity files from both studies were loaded using the *Minfi* library [16]. We then calculated β values, which range from 0 (completely unmethylated CpG) to 1 (completely methylated). We continued applying a series of quality controls (QCs) at the sample and probe level. Briefly, we excluded not detected probes (detection *p*-values > 0.05 or beadcount lower than 5 in 5% of samples) or that were located at allosomal, multihit or polymorphic positions. Regarding samples, we excluded those showing sex mismatch or a low call rate (less than 98%). For each batch we normalized the β matrix using the beta-mixture quantile normalization method and merged these batches into two strata: discovery and replication samples [17]. For each stratum we corrected the batch effect and adjusted for the estimated cellular counts (see more details in supplementary methods). After this set of QCs, we ended having a discovery sample composed of 316 individuals and 370,344 CpGs (supplementary Table S1), while in replication phase we had 92 individuals and 358,834 CpGs.

### Gene expression quantification

Eighteen individuals from the BasicMar cohort, fulfilling the same stringent selection criteria as stated previously, were assessed for whole transcriptome expression in peripheral blood samples collected in PAXgene tubes (Qiagen) at 6 h, 24 h and 3 month post-stroke and stored at -80ºC until further use. Briefly, total RNA was isolated using the PAXgene Blood RNA extraction Kit (Qiagen) and analyzed with the GeneChip Human Gene 2.0 ST (Affymetrix) at the Microarray Analysis Service of Hospital del Mar Research Institute with Ovation WB Solution commercial kit (NuGEN). After quantity and quality check, mRNA samples were converted to complementary DNA (cDNA), labeled, hybridized, washed and scanned to generate CEL data files, following standard protocols. Importantly, in seven of these patients we also had DNAm data.

### Statistical analysis and bioinformatics

#### Descriptive analyses

Data were expressed as mean (± standard deviation), median (interquartile range) or count (percentage) according to the type and distribution of each variable. Main demographic, clinical and outcome variables were compared between patients with good (mRS 0 to 2) and poor prognosis (mRS 3 to 6) using *t*-, U Mann–Whitney or χ^2^ tests, as appropriate. To know which of these factors were independently associated with stroke outcome we built a logistic regression model using a forward-stepwise algorithm based on Akaike information criterion (AIC).

#### Differentially methylation positions

We firstly explored whether patients with poor stroke outcome presented DMPs. To that aim, we first build linear models in the discovery sample in which methylation at the CpG level was the dependent variable and dichotomized mRS was the independent variable of interest. This set of models were additionally adjusted for: age, sex, smoking habit, type 2 diabetes (DM2), hypertension (HT), dyslipidemia, NIHSS at 24 h and previous mRS. These covariables were selected based on previous analyses (see Sect. "Descriptive analyses": previous mRS, NIHSS at 24 h and age) or previous literature reporting associations between vascular risk factors and DNAm (smoking, HT, DM2 and dyslipidemia) [18, 19]. We used 24-h NIHSS instead of initial NIHSS to account for the effect of treatment on stroke prognosis. We set α value at 0.05 and epigenome-wide significance at 10^–7^ (Bonferroni adjustment). Nominal significance was set at 10^–5^, and those CpGs under this cutoff were considered as candidates to be replicated. Besides, we conducted a bootstrap to test the robustness of our results in the discovery study and a Bayesian method to correct *p*-values for statistic inflation (*Bacon* library, see the supplementary methods) [20]. By applying these adjustments, we accounted for two of the main sources of bias in EWAS: influential cases and test-statistic inflation. Finally, we checked whether the relationship between stroke outcome and DNAm was moderated by relevant variables such as blood cell fractions and stroke subtype (see the supplementary methods).

Candidates of interest (*p*-value < 10^–5^) were then tested in the replication cohort. Models were constructed adjusting for the same set of variables as in the discovery study. All results were adjusted for multiple testing at this stage (false discovery rate, Benjamini & Hochberg method). Finally, all candidates were annotated using the Illumina manifest data and the GREAT software [21]. Additionally, we checked the methylation levels by mRS levels (from 0 to 5–6) of CpGs that were nominally significant at both stages to infer whether these DMPs were ordinally associated with mRS.

We additionally combined full epigenome-wide results coming from both cohorts using the METAL software [22]. We used a fixed effects model weighted by the number of subjects from each cohort. Epigenome-wide and nominal significance levels were set using the same cutoffs as described above. We considered a candidate as replicated when Q-value was lower than 0.05 in the replication phase and meta-analysis *p*-value was significant at the epigenome-wide level (10^–7^) [12].

#### Post-EWAS analyses

We compared the longitudinal expression profiles (at 6 h, 24 h and 3 months) between patients with good and poor outcomes for the genes annotated to the replicated CpGs. This analysis aimed to confirm whether DMPs corresponded to changes in gene expression levels. See the supplementary methods for additional details on the statistical analysis.

In addition, using the meta-analyzed *p*-values, we searched for differentially methylated regions and biological pathways in patients with poor outcome as described in the supplemental methods.

## Results

### Descriptive analysis

#### Discovery sample

We included 316 patients in the discovery study (Fig. 1). Patients had a median age of 77.5 years (Q_1_-Q_3_ = 69.0 to 83.0) and 166 (52.5%) had a poor stroke outcome (mRS > 2) at 3 months after stroke onset, as summarized in Table 1. In the univariate analyses, patients with poor prognosis were older, more likely to be men and had a higher initial and 24-h stroke severity (Table 1). When we built a multivariate logistic regression model, NIHSS at 24 h, previous mRS and age remained as independent factors significantly associated with poor prognosis (supplementary Table S2).

**Table 1:** Main characteristics of the discovery sample

	Whole sample (*N* = 316)	Good outcome (*N* = 150)	Poor outcome (*N* = 166)	*p*-value
Age, years	77.5 (69.0;83.0)	76.0 (65.0;81.0)	79.0 (71.2;84.0)	0.001
Sex, female	152 (48.1%)	62 (41.3%)	90 (54.2%)	0.030
Hypertension	236 (74.7%)	109 (72.7%)	127 (76.5%)	0.513
Dyslipidemia	138 (43.8%)	71 (47.7%)	67 (40.4%)	0.235
Diabetes	129 (40.8%)	64 (42.7%)	65 (39.2%)	0.604
Atrial fibrillation	176 (55.7%)	73 (48.7%)	103 (62.0%)	0.023
Smoking	85 (26.9%)	48 (32.0%)	37 (22.3%)	0.069
Toast				0.016
Atherothrombotic	108 (34.2%)	56 (37.3%)	52 (31.3%)	
Cardioembolic	184 (58.2%)	77 (51.3%)	107 (64.5%)	
Undetermined	24 (7.60%)	17 (11.33%)	7 (4.22%)	
Baseline NIHSS	8.0 (5.0;17.0)	5.0 (4.0;8.0)	15.0 (7.0;19.0)	< 0.001
NIHSS 24 h	7.0 (4.0;14.0)	4.0 (2.0;6.0)	13.0 (7.0;19.0)	< 0.001
rTPA treatment	77 (24.5%)	36 (24.2%)	41 (24.8%)	0.992
Previous mRS				< 0.001
0	224 (70.9%)	127 (84.7%)	97 (58.4%)	
1	45 (14.2%)	14 (9.33%)	31 (18.7%)	
2	47 (14.9%)	9 (6.00%)	38 (22.9%)	
3-month mRS				–
0	46 (14.6%)	–	–	
1	46 (14.6%)	–	–	
2	58 (18.4%)	–	–	
3	47 (14.9%)	–	–	
4	47 (14.9%)	–	–	
5	6 (1.90%)	–	–	
6	66 (20.9%)	–	–	

Values represent frequencies (percentage) or medians (interquartile range) in the whole replication sample and by stroke outcome. Last column shows which variables are significantly associated with stroke outcome

Keywords: mRS, modified Rankin scale; NIHSS, National Institute of Health Stroke Scale; TPA, tissue plasminogen activator

#### Replication sample

We recruited 92 participants for the replication stage (Fig. 1). Main characteristics of these subjects can be found in supplementary Table S3. There were 55 (59.8%) participants with a poor stroke outcome, and average age was 76.5 (Q_1_-Q_3_ = 69.0 to 81.0). Variables associated with poor stroke outcome were similar as those observed in the discovery phase (Table 1 & supplementary Table S3). When we compared both cohorts, we only observed that patients in the discovery sample had a higher prevalence of hypertension (discovery vs replication: 236 [74.7%] vs 54 [58.7%]), diabetes mellitus (129 [40.8%] vs 15 [16.3%]) and were more likely to be active smokers (85 [26.9%] vs 12 [13%]). We observed no differences between cohorts in terms of stroke prognosis, demographic variables, other vascular risk factors and stroke severity (*p*-value > 0.05 in all cases).

### Differentially methylated positions

#### Discovery stage

**Fig. 2:**
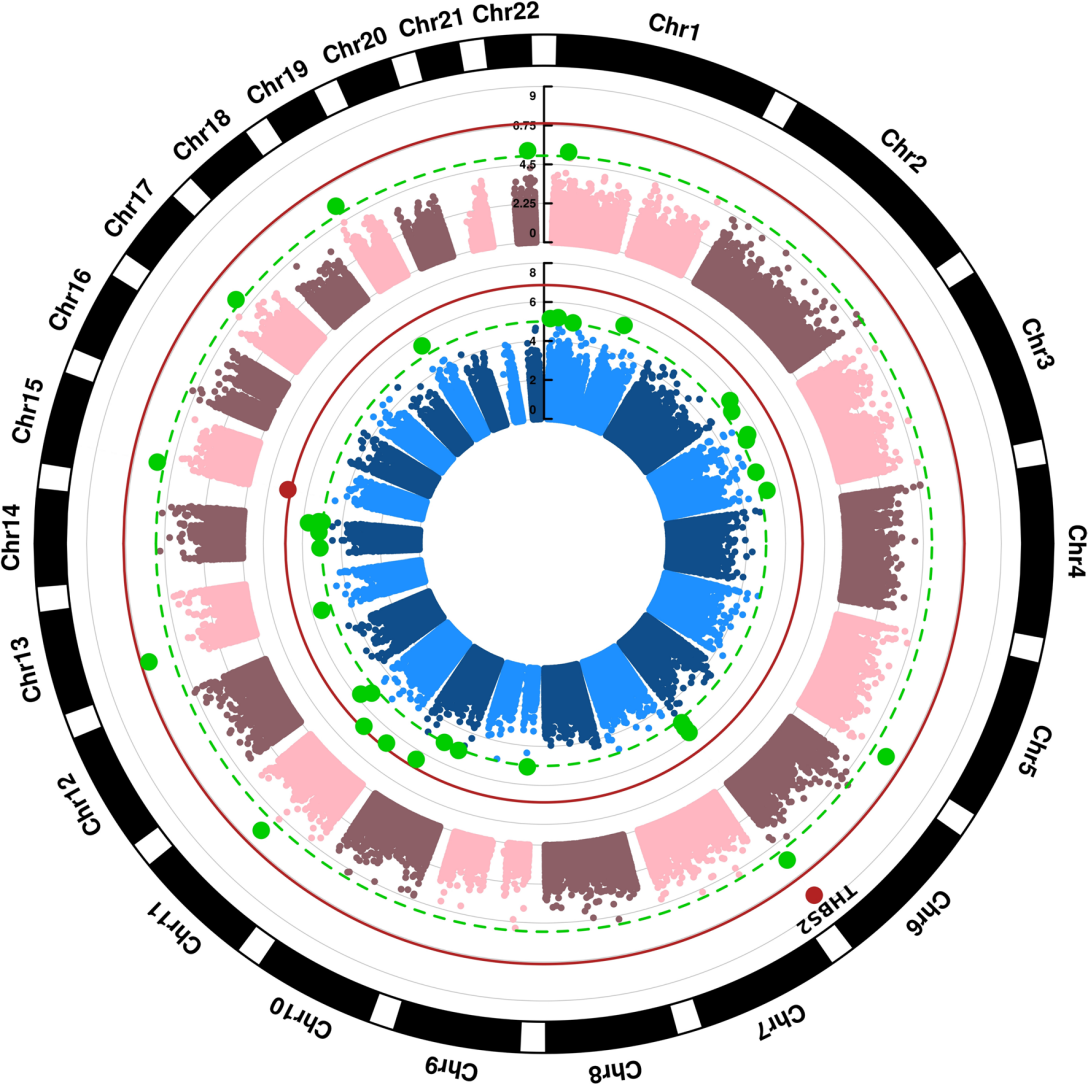
Epigenome-wide association study of stroke outcome. Manhattan plots. The blue and pink Manhattan plots correspond to the discovery and meta-analysis stages, respectively. Red solid line represents the cutoff for epigenome-wide significance, while the green dashed line for nominal significance (10^–5^). The annotated hit (THBS2) was validated in the replication study (*p*-value < 0.05)

**Fig. 3:**
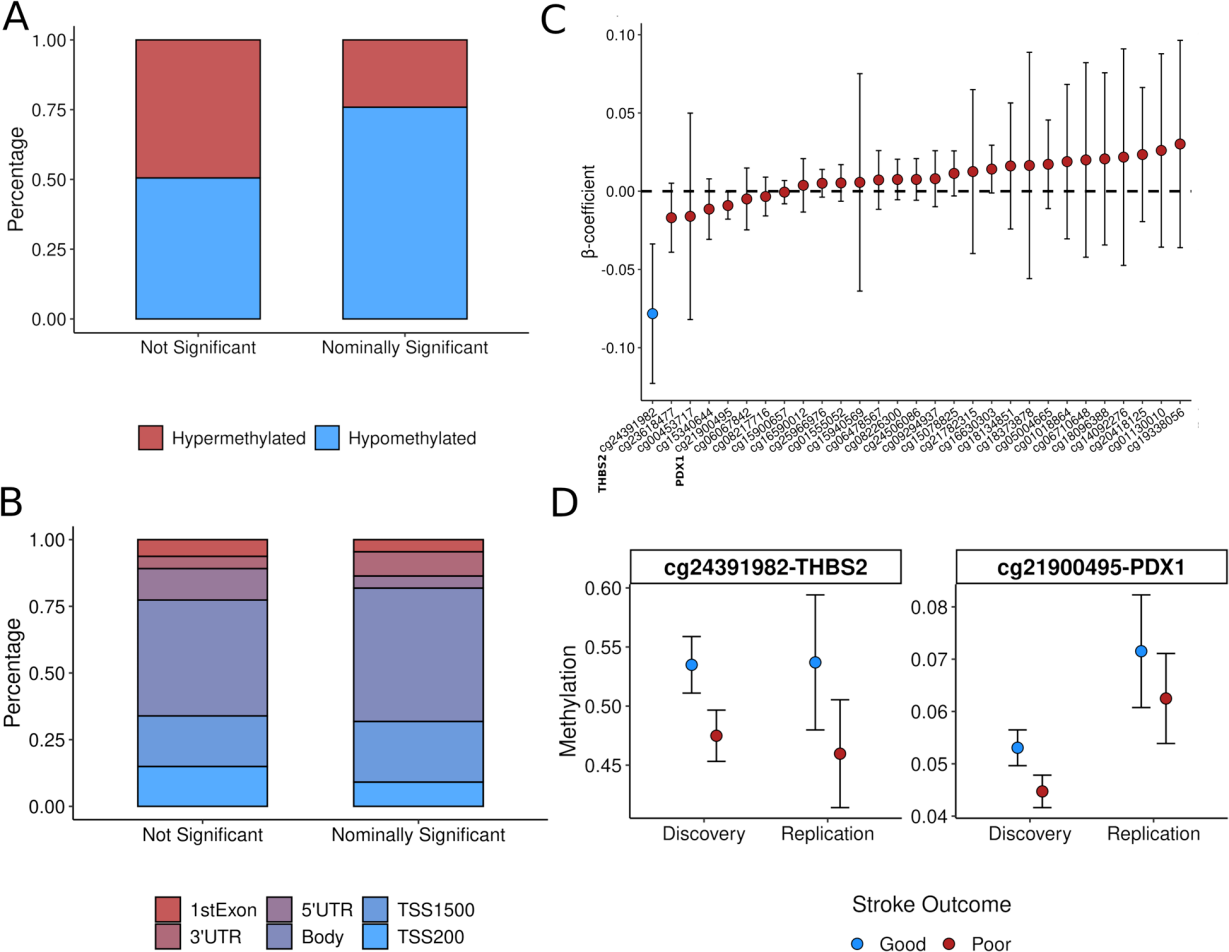
Differentially methylated positions. **A** Bar plots showing the proportion of hyper- and hypomethylated CpGs in patients with poor outcome by CpG statistical significance (10^–5^). **B** Localization of CpGs by statistical significance (10^–5^). **C** Results obtained in the replication phase in the subset of nominally significant CpG. Dots correspond to β-coefficients and error bars to 95% confidence interval. Blue CpGs are those significant candidates after correcting for multiple testing. **D** Dots represent marginal means of multivariate models interrogating the association between methylation at these sites and stroke outcome for both discovery and replication studies. Error bars correspond to 99% confidence interval

After applying quality controls, we tested the association between 370,344 CpGs and stroke outcome (good vs poor outcome) in the discovery sample (supplementary Table S1). We found 29 nominally significant CpGs (*p*-value < 10^–5^), which were considered as candidates to be replicated (Fig. 2 & supplementary Table S4), most of them being hypomethylated in patients with poor outcome (Fig. 3A). Moreover, nominally significant CpGs were more frequently located within the gene body compared to non-significant CpGs (Fig. 3B). Only one of these CpGs, annotated at the promotor region of Gamma-Aminobutyric Acid Type A receptor subunit beta 3 gene (GABRB3), was significant at the genome-wide level (*p*-value < 10^–7^, Fig. 2 & supplementary Table S4). Differences in methylation between patients with good and poor prognosis in this set of 29 nominally significant CpGs are displayed in supplementary Figure S1.

To correct for the influence of outlier observations, we ran a bootstrap in nominally associated CpGs (*N* = 29) and all of them remained significant (supplementary Figure S2). On the other hand, when we corrected our results by test-statistic inflation we found that only 6 out of 29 CpGs remained nominally significant (*p*-value < 10^–5^) after the adjustment (supplementary Table S4 & supplementary Figure S3).

We also checked whether the relationship between methylation at these 29 CpG sites and stroke outcome was moderated by blood cell factions, finding that only 2 CpGs (cg14092276 & cg06710648) were differentially hypomethylated only in granulocyte cells (supplementary Table S5). Finally, none of these 29 candidates CpGs showed a significant interaction with stroke subtype (supplementary Table S6), which suggests that differences in DNA methylation between patients with good and poor prognosis were constant across stroke etiologies.

#### Replication stage

The 29 candidate DMPs from the discovery stage were tested for association with poor stroke outcome in the replication sample. Two of these CpGs, cg24391982 (thrombospondin-2 [*THBS2*] gene) and cg21900495 (insulin promoter factor 1 [*PDX1*]), were nominally significant (*p*-value < 0.05), and the direction of the effect was consistent with that observed in the discovery stage (Fig. 3C & supplementary Table S4). However, only cg24391982 (*THBS2*) was significant after correcting for multiple testing in the replication stage (false discovery rate, Q-value < 0.05; Fig. 3C & supplementary Table S4). Both candidates were hypomethylated in patients with poor outcome and were located at the gene body according to the Illumina manifest. Differences in methylation signal between patients with good and poor prognosis for both candidates and both stages are represented in Fig. 3D. In supplementary Figure S4 we show the methylation signal at these CpGs for each value of the mRS, observing similar results.

#### *Meta*-analysis

Discovery and replication results were finally meta-analyzed in a fixed effect model, and we found that cg24391982 (*THBS2*) was significant at the genome-wide level (*p*-value = 6.39·10^–9^), while cg21900495 was only nominally significant (*p*-value = 4.0·10^–7^, Fig. 2 & supplementary Table S4). We also found 10 DMPs at nominal significance (*p-*value < 10^–5^; supplementary Table S7). One of these DMPs (cg16805094) was also annotated to THBS2 gene, and in supplementary Figure S5 we show the meta-analyzed *p-*values obtained in the full region of this gene.

#### Expression analyses

**Fig. 4:**
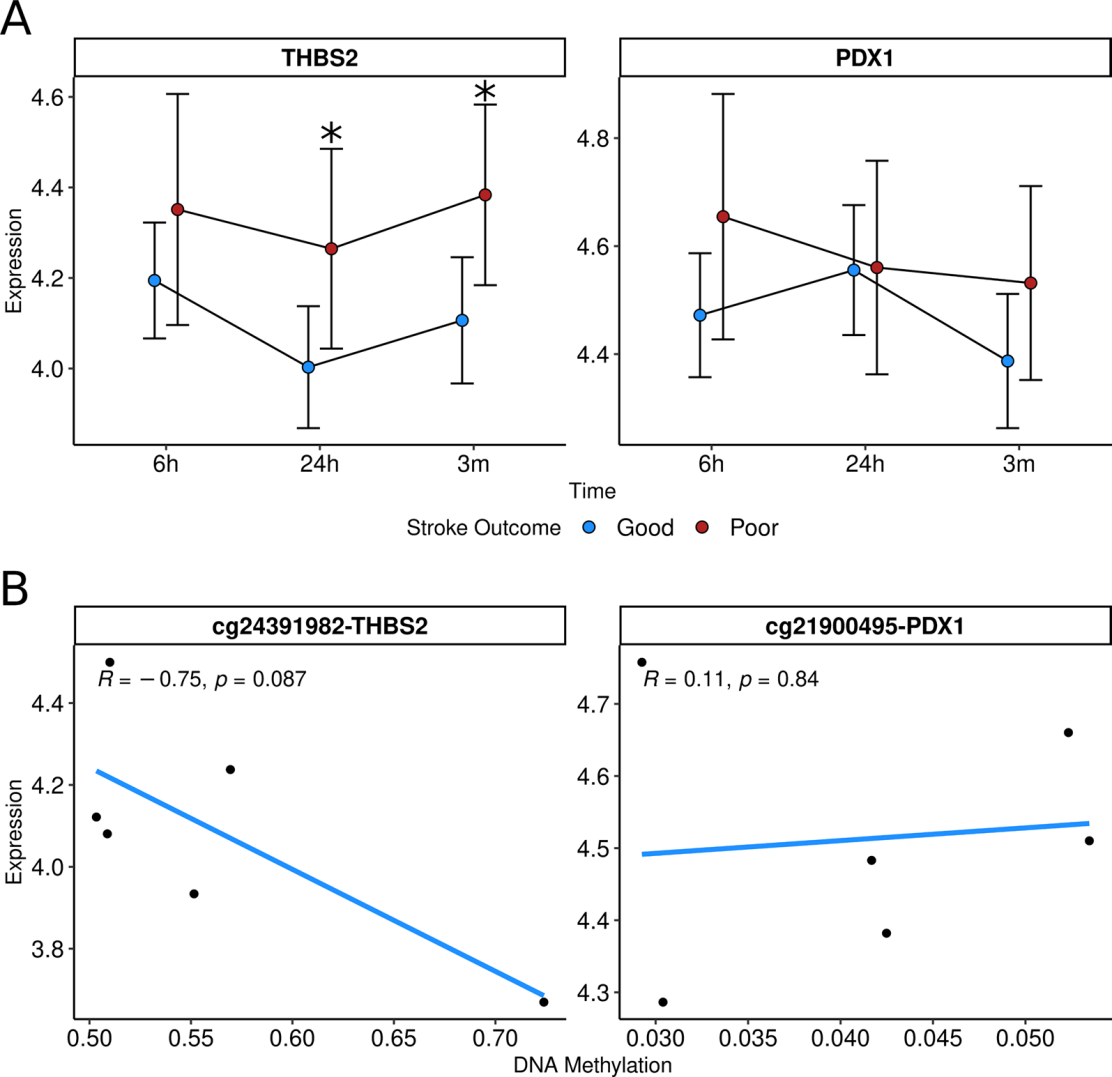
Gene expression analysis. Longitudinal expression of genes annotated to CpGs that were replicated. **A** THBS1 and PDX1 expression levels at 6 h, 24 h and 3 months post-stroke in patients with good (blue, *N* = 13) and poor (red, *N* = 5) outcome. Models were adjusted for age and sex. **B** Correlation between DNAm and gene expression (*N* = 7). *p-value ≤ 0.05

We also studied the longitudinal expression of THBS2 in a small sample with gene expression data comprising 13 and 5 patients with good (72.2%) and poor prognosis (28.8%), respectively (*N* = 18, supplementary Table S8). There was no significant main effect of time on THBS2 expression (*p*-value = 0.068). Similarly, the interaction between time and stroke outcome was not statistically significant (*p*-value = 0.753), suggesting that patients with poor and good outcomes followed similar trajectories over time. However, patients with poor outcomes showed a higher expression of THBS2 at 24 h and 3 months post-stroke (*p*-value < 0.05, Fig. 4A). We also conducted this analysis for *PDX1,* which was only nominally significant, finding no significant results (Fig. 4A).

In seven individuals, we had both DNAm and gene expression data. Figure 4B shows the correlation between DNAm at cg24391982 and the expression of the THBS2 gene. Although the correlation was not statistically significant, we observed a trend toward significance, indicating that hypermethylation was associated with THBS2 silencing (r = -0.75, *p*-value = 0.087). Additionally, we analyzed the correlation between DNAm at cg21900495 and the expression of the PDX1 gene, finding no significant results (Fig. 4B).

### Differentially methylated regions

**Fig. 5:**
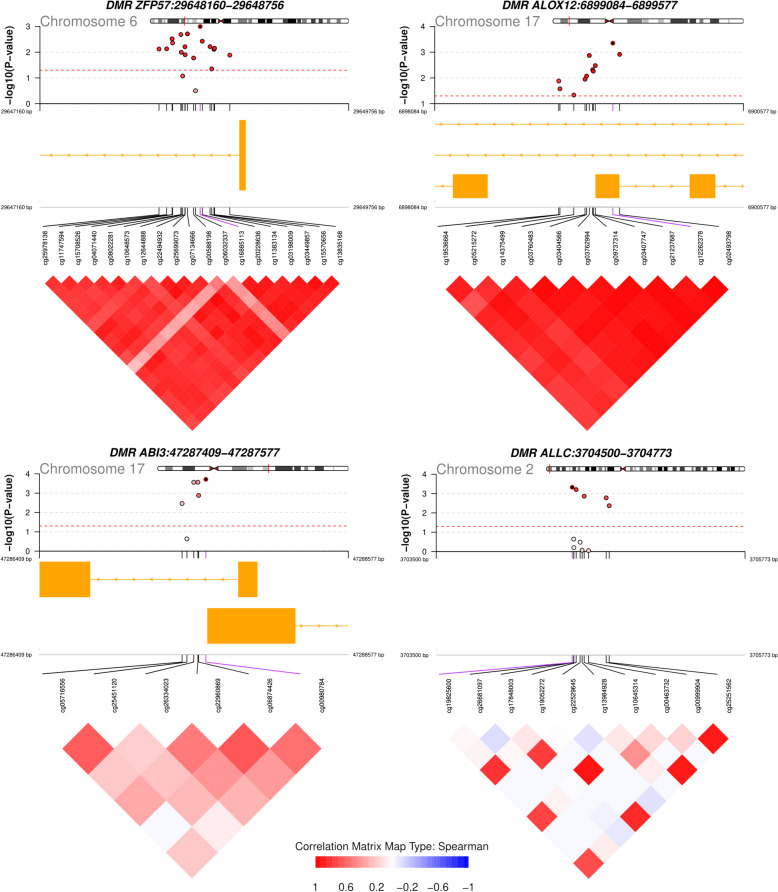
Differentially methylated regions in patients with poor stroke outcome. Region plots showing the four significant DMRs associated with stroke outcome in the meta-analysis (Q-value < 0.05). Each dot represents one CpG conforming the region. The Y-axis corresponds to the -log10 *p*-value and the X-axis to the CpG location. The dot color corresponds to the correlation of each CpG with the most significant CpG of each region (red indicates a positive correlation, while blue an inverse correlation). At the bottom of each plot there is the correlation matrix of the methylation levels of those CpGs conforming the region

We explored whether existed differentially methylated regions (DMRs) using the meta-analyzed *p*-values as input. We show the significant DMRs in supplementary Table S9. After correcting for multiple testing, we found 4 DMRs that were annotated to the following genes: zinc finger protein 57 homolog (*ZFP57*, 19 CpGs), Arachidonate 12-Lipoxygenase 12S Type (*ALOX12*, 11 CpGs), ABI Family Member 3 (*ABI3*, 6 CpGs) and Allantoicase (*ALLC*, 5 CpGs). We also found one DMR that was marginally significant (Q-value = 0.052), which was annotated to the gene Homeobox A5 (*HOXA5*, 8 CpGs). In Fig. 5 we show the locus plots for these significant DMRs (Q-value < 0.05). As expected, CpGs that conformed each region were positively correlated. DMRs annotated to *ZFP57*, *ALOX12* and *ABI3* were located at the gene body, while *ALLC* was an intergenic region.

### Gene set enrichment analysis

We observed no significant gene set enrichment in the meta-analyses results after adjusting for multiple testing. In supplementary Figure S6 we show top candidate gene sets for Gene-Ontologies and Reactome (Q-value < 0.25). Among top candidates of Reactome database, we found interleukin-27 signaling (*p*-value = 1.0·10^–4^) and presynaptic function of Kainate receptors (*p*-value = 4.7·10^–4^), but none of these was significant after adjusting for multiple testing (supplementary Figure S6).

## Discussion

This is the first study that explored how whole blood DNAm measured during the acute phase conditions the functional status at 3 months after stroke onset. The study consisted of a two-phase discovery and replication studies and a meta-analysis. It reports that hypomethylation at CpG cg24391982 (*THBS2*) is associated with poor stroke outcome (mRS > 2). Besides, our study also identified several DMRs annotated to the following genes: *ZFP57*, *ALOX12*, *ABI3*, *ALLC*.

The most consistent finding in our study is the association between methylation at *THBS2* gene and stroke outcome. *THBS2*, or alternatively *TSP-2*, is a member of the thrombospondin subgroup A family, together with thrombospondin-1 (*THBS1*), which are angiostatic factors involved in angiogenesis inhibition, but also in synaptogenesis and cell–matrix interactions [23]. Previous studies in humans reported that this protein shows specific temporal profiles within the acute phase, being upregulated in patients with stroke as compared to controls [24]. Other studies conducted in patients at increased cardiovascular risk found that higher *THBS2* expression was related to cardiovascular mortality and adverse cardiovascular events [25]. Similarly, levels of *THBS1* have been linked to adverse outcomes and complications after aneurysmal subarachnoid hemorrhage [26]. In our study we observed that patients with a poor stroke outcome showed lower methylation levels as compared to patients with good prognosis. In general terms, hypomethylation is associated with increased gene expression, but this might depend on the localization of the CpG respective to the gene, so we cannot draw a causal conclusion [10]. When we studied gene expression, we found that patients with poor outcome showed a higher *THBS2* expression at 24 h and 3 months after stroke onset, in line with the previous literature. However, our gene expression data were limited to a small group of participants. Further larger studies tracking patients throughout the entire acute phase and collecting data on both DNA methylation and THBS2 expression will provide better insight into the role of this gene in stroke prognosis and its potential as a therapeutic target.

Another interesting DMP was annotated to *PDX1* gene, which is a transcriptional activator at several genes, including insulin, somatostatin and glucose transporter type 2 [27]. Variants in this gene have been associated with increased risk of diabetes mellitus and hypertension [27, 28]. Both hyperglycemia and elevated blood pressure have been associated with a worse recovery after stroke, which might explain the link between methylation at *PDX1* gene and poor stroke outcome in our study [29, 30]. However, results about this locus should be interpreted with caution because this DMP was only nominally significant. Moreover, we observed no differences in PDX1 expression between patients with poor and good outcome.

We also found several DMRs significantly associated with stroke outcome. One of them was annotated to the *ALOX12* gene, a member of the lipoxygenase enzymes family, which are implicated in both pro- and anti-atherogenic processes [31]. Kim et al. (2020) found that *ALOX12* displayed higher methylation levels in plaques than in non-plaque intima [32], in line with results from Portilla-Fernández and collaborators (2020), who reported a DMR at *ALOX12* gene associated with carotid intima media thickness [33]. Carotid intima media thickness, in turn, is known to predict 3-month outcome as measured by mRS [34], linking our results with those relating atherogenesis to *ALOX12* activity.

We also observed 3 additional DMRs annotated to *ZFP57*, *ALLC*, *ABI3*, and all of them have been described to be involved in Alzheimer’s disease (AD) [35–38]. For instance, hypermethylation at *ALLC* has been associated with advanced Braak stages [35, 36]. Similarly, Li et al. (2021) described a DMR at gene *ZFP57* in patients with AD showing a clinical progression within a follow-up. Finally, rare coding variants in *ABI3*, a gene highly expressed in microglia, were associated with increased risk of AD in a case–control study [38]. Cognitive impairment is one of the main consequences of stroke, such that 20 to 30% of stroke survivors show some degree of cognitive impairment [39]. Our results might suggest that methylation at these specific sites might be contributing to a worse stroke outcome via a decline in cognitive function.

As strengths of the study, it is worth highlighting first its robust design, based on two-phases, discovery and replication studies followed by a meta-analysis. Secondly, the solid selection criteria in the discovery sample are nested in a cohort of well-phenotyped stroke participants [9]. It has been also considered step-by-step several sources of bias in epigenomic studies, such as the influence by outlier observations, results inflation or the effect of confounding variables, and finally, the inclusion of several epigenetic approaches such as studying DMPs, DMRs and functional enrichment. Moreover, we studied the gene expression of genes annotated to replicated DMPs in a small group of participants, offering further insight into the biological significance of these DMPs.

As limitations, we could not exclude patients based on previous mRS or stroke location in the replication study, which might have added noise to the replication results, even if these participants seemed to be healthier as compared to the discovery sample. Despite the fact that it is the largest study so far to address this issue with such rigorous phenotyping and selection criteria, the sample size could be insufficient for detecting other less intense associations, especially at the replication stage. However, these two limitations could prevent finding other new associations that require higher statistical power, but do not diminish the significance or robustness of the reported associations. Moreover, our study reports associations but does not establish causality. Further experimental studies will be needed to investigate this aspect. Another recurrent limitation in epigenetic studies is the use of whole-blood samples to estimate DNAm. Although it has been reported a good correlation between whole-blood and cerebral DNA methylation in some biological processes, it is true that some specific brain-tissue mechanisms may not be detected in blood. However, there are no practical means to study brain-tissue samples during the acute phase of patients who survive a stroke event. Finally, this study involved only Caucasian individuals, and our results might not generalize to other ethnicities. Future international collaborative efforts are needed to conduct a trans-ethnic EWAS of stroke outcome.

## Conclusions

Methylation at *THBS2* gene, involved in angiogenesis, is associated with poor stroke outcome at 3 months. Furthermore, the regions analysis revealed four DMRs annotated to genes previously related to atherogenesis and cognitive impairment. These findings suggest an association between DNAm and stroke outcome, which might help to identify new stroke recovery mechanisms.

### Supplementary Information

Supplementary information 1 (Docx 1439 Kb)

## Data Availability

Data will be shared upon reasonable request from qualified researchers.
