# Supplementary material for: DNA methylation and stroke prognosis: an epigenome-wide association study
Source: Clin Epigenetics. 2024 Jun 6;16:75. doi: 10.1186/s13148-024-01690-2 (PMC11155152; doi:10.1186/s13148-024-01690-2)
Supplement: Supplementary file 1 — Supplementary information 1 (Docx 1439 Kb) [file 13148_2024_1690_MOESM1_ESM.docx]

**CONTENT**

[Supplemental Methods 3](#_Toc167700340)

[Setting and participants 3](#_Toc167700341)

[Discovery Sample 3](#_Toc167700342)

[Replication Sample 4](#_Toc167700343)

[Vascular risk factor variables 4](#_Toc167700344)

[DNA methylation quantification 4](#_Toc167700345)

[Epigenome-Wide Association Study (EWAS) QC 5](#_Toc167700346)

[Gene expression QC 6](#_Toc167700347)

[Statistical Analyses & Bioinformatics 6](#_Toc167700348)

[Bootstrap and statistic-inflation 6](#_Toc167700349)

[Potential moderation effects 7](#_Toc167700350)

[Gene Expression Analyses 7](#_Toc167700351)

[Differential Methylation Regions (DMR) analyses. 8](#_Toc167700352)

[Biological pathways analyses 8](#_Toc167700353)

[Data Sharing 8](#_Toc167700354)

[Bioinformatic Resources 8](#_Toc167700355)

[Resource table 9](#_Toc167700356)

[References methods 9](#_Toc167700357)

[Supplemental Figures 12](#_Toc167700358)

[Supplementary Figure S1. 12](#_Toc167700359)

[Supplementary Figure S2. 13](#_Toc167700360)

[Supplementary Figure S3. 14](#_Toc167700361)

[Supplementary Figure S4. 15](#_Toc167700362)

[Supplementary Figure S5. 16](#_Toc167700363)

[Supplementary Figure S6. 17](#_Toc167700364)

[Supplemental tables 18](#_Toc167700365)

[Supplementary Table S1 18](#_Toc167700366)

[Supplementary Table S2 20](#_Toc167700367)

[Supplementary Table S3 21](#_Toc167700368)

[Supplementary Table S4 22](#_Toc167700369)

[Supplementary Table S5 24](#_Toc167700370)

[Supplementary Table S6 28](#_Toc167700371)

[Supplementary Table S7 29](#_Toc167700372)

[Supplementary Table S8 30](#_Toc167700373)

[Supplementary Table S9 31](#_Toc167700374)

# Supplemental Methods

## Setting and participants

### Discovery Sample

The study participants were well-characterized IS cases coming from 2 cohorts with data about DNA methylation (BasicMar-1 [N=619] & BasicMar-2 [N=380]) of European-ancestry nested in the BASICMAR Register (consecutive patients assessed in Hospital del Mar from 2009-2018 with diagnosis of stroke).

Patients were then selected based on the following inclusion criteria (GODs project)[1]: (1) Caucasian individuals older than 18; (2) Acute anterior ischemic stroke patients, assessed by a neurologist and confirmed in brain imaging with computed tomography or magnetic resonance; (3) Functional independent status before the stroke, defined as modified Rankin Scale (mRS) < 3; (4) Initial stroke severity >2, according to the National Institute of Health Stroke Scale (NIHSS); (5) Information on post-stroke functional status at 3 months; (6) Availability of DNA samples.

Exclusion criteria were: (1) Posterior vascular territory stroke; (2) lacunar or “Other determined etiology” (unusual etiology) stroke subtype according to Trial of ORG 10172 in Acute Stroke Treatment (TOAST) classification[2]; and (3) recurrence of stroke during the follow-up period; (4) Presence of concomitant pathology that may compromise the short term survival of the individual or may difficult the results interpretation.

We excluded lacunar and posterior territory strokes from our analysis because they exhibit a weak correlation between the size of the infarction and clinical symptoms[1]. In such cases, the processes of recovery and tissue regeneration mechanisms may be confounded or obscured by the random location effect.

Therefore, from the original 999 stroke subjects with epigenetic data and stroke outcome information, a total number of 323 subjects met these strict inclusion and exclusion criteria (Figure 1, discovery sample).

### Replication Sample

Replication sample consisted in a subset of 92 patients who belong to the St. Pau cohort (Neurovascular Genetics and Pharmacogenomics lab, Institut d’Investigacio Biomedica i Recerca de l’Hospital de Sant Pau, Barcelona, Spain), who had DNA methylation (DNAm) data and met selection criteria (St. Pau-1 [N=29] & St. Pau-2 [N=63]). However, in this case we lacked the following data: (1) previous functional status; (2) whether the infarct was posterior or anterior. Therefore, patients were not rule-out based on these criteria.

## Vascular risk factor variables

Clinical and epidemiological data were obtained during the stroke acute phase, hospitalization and at the 3 months follow-up visit as described in GODs project[1]. Vascular risk factors, sex and age were registered in a structured questionnaire for every individual via patient or proxy interview, and medical records review.

Vascular risk factors were coded according to the definitions of international guidelines as follows: hypertension (evidence of at least 2 raised blood pressure measurements, systolic >140 mmHg or diastolic >90 mmHg, recorded on different days before stroke onset, or use of an anti-hypertensive medication), diabetes (at least two time of admission serum fasting blood glucose levels ≥126 mg/dL or use of diabetes medication), hyperlipidemia (serum cholesterol concentration >220 mg/dl, or serum triglyceride concentration >150 mg/dl, or use of medication prescribed to control), coronary artery disease (documented history of angina pectoris or myocardial infarction), atrial fibrillation (documented history or diagnosis during hospitalization), prior stroke (history of prior ischemic stroke). Smoking habit was self-reported and classified in two categories: smokers (smokers or gave up smoking <5 year before stroke), and no smokers (never smoked or gave up smoking >5 years before stroke).

## DNA methylation quantification

*DNA extraction*

We used three different systems to extract the DNA in the BasicMar cohort: the Chemagic Magnetic Separation Module I system (Chemagen), the Autopure LS (Qiagen) and the Gentra Puregene Blood kit (Qiagen, Hilden, Germany). On the other hand, in the replication cohort (St Pau) we only used the Gentra Puregene Blood kit (Qiagen, Hilden, Germany). DNA extractions were stored at minus 20ºC and the quality was checked with nanodrop.

*Discovery Sample*

For the BasicMar study, genome-wide DNAm was obtained in three technical runs. The first two runs were analyzed using the Human Methylation 450K Beadchip (485,577 analyzed CpGs), conforming the BasicMar-1 cohort (N=252). BasicMar-2 (N=71) consisted in a third technical run in which we used the Infinium Methylation EPIC beadchip consisting of 865,918 probes (Illumina, Netherlands, Eindhoven). Manufacturer’s protocols were strictly followed, and all runs were scanned using the Illumina HiScan SQ scanner at Progenika Biopharma in Bizkaia, Spain. Therefore, a total of 323 subjects were included in the Discovery phase before quality controls as showed in Figure 1.

*Replication Study*

Samples from the St. Pau cohort were analyzed in 4 technical runs, 29 using the 450K Beadchip array and 63 using the EPIC array. DNA methylation intensities were determined using the GenomeStudio Software (Illumina).

## Epigenome-Wide Association Study (EWAS) QC

Intensity files from both studies were loaded using the R-libraries Minfi and ChAMP[3,4]. We then calculated β values, which range from 0 (completely unmethylated CpG) to 1 (completely methylated) and correspond to the ratio between methylated intensity and total intensity.

Regarding sample quality controls (QCs), we removed those samples presenting sex mismatch (Minfi algorithm)[5], call rate lower than 98%, that represented outlier observations or that had missing covariables. Therefore, in the discovery sample 2 subjects were excluded from the first technical run, 4 subjects from the second one and 1 subject from the third one. This left a total of 316 individuals for the discovery sample after QCs (supplementary Table S1). In the replication cohort 5 samples were excluded due to sex mismatch and 1 sample due to failed typing, leaving a total of 92 samples.

Regarding probes QCs, we considered that a CpG was not detected when 1% of samples showed a detection p-value > 0.05 or when beadcount was lower than 5 in 5% of subjects. Besides, we excluded those CpGs corresponding to SNP positions[4], that show cross-reactivity, or that belong to non-autosomal chromosomes. We subsequently normalized β values from each batch using the beta-mixture quantile normalization method[6]. After doing these QCs, in the discovery sample we merged the technical runs in one file which yield a total of 370,344 CpGs (common CpGs between 450K and EPIC chips). The same set of QCs were followed in the replication cohort, leaving a total of 358,834 CpGs for analysis.

In both samples (discovery and replication) we studied the batch effect produced by technical runs by first reducing the DNAm dataset dimensions via singular-value decomposition (SVD), and then plotting the first dimension against the second one. We subsequently removed the batch effect using the sva library[7]. Finally, we calculated the estimated white cell counts from DNAm using the Houseman method and regressed out their effect on the β-values matrix[8].

## Gene expression QC

We explored whether genes annotated to replicated CpG candidates (THBS2 and PDX1) were differentially expressed in patients with poor outcome. To that aim, we measured the gene expression in 18 individuals fulfilling the same strict inclusion criteria, as explained in section 2.5.

The quality controls of raw data were assessed using the Expression Console (Affymetrix), which includes processing and hybridization controls within the array, as well as the standardization and log2 transformation of the intensity signals. Data normalization was run in R using the robust multi-array average method (RMA). All the transcripts with SD>30% were excluded.

## Statistical Analyses & Bioinformatics

### Bootstrap and statistic-inflation

We conducted a bootstrap to test the robustness of our results in the discovery study. Briefly, significant candidates at nominal *p*-value (10^-5^) were iteratively tested in 10,000 resampled sets of the original cohort. From each candidate, we acquired a distribution of β coefficients, and we calculated the 0.1 and 99.9 percentiles of this distribution (α=0.05/n, where ‘n’ represents the number of candidates). Bootstrap analysis helped to check whether results were influenced by outlier observation or influential cases. On the other hand, as test-statistic inflation might lead to overestimation of results, we applied a Bayesian method to correct *p*-values (*Bacon* library)[9].

### Potential moderation effects

We investigated whether the relationship between stroke outcome and CpG candidates (10^-5^) was influenced by cell heterogeneity. To do this, we used the β matrix obtained prior to any adjustments for cell types and examined the interaction between stroke outcome and cell fractions using the *EpiDISH* library[8,10]. This analysis provided insight on whether the relationship between the phenotype and methylation was observed only in specific cellular populations. Additionally, we also checked whether the stroke subtype moderated the effect of stroke outcome on DNAm. Therefore, we repeated the same general linear models described in section 2.6.2 of the main document, but including a term for the interaction between stroke etiology (TOAST) and outcome. Results for the moderation effects have been corrected by multiple testing (false discovery rate).

### Gene Expression Analyses

We longitudinally compared the expression of two transcripts, annotated to THBS2 (AffyID: 17025844) and PDX1 (AffyID: 16773547), at 6 hours, 24 hours, and 3 months between patients with poor and good outcomes. We constructed linear mixed models, with the expression levels of THBS2 or PDX1 as the dependent variable, and age, sex, and time as covariates in the baseline model. We did not include other covariates given the small sample size. This model also included random intercepts for patient ID.

To determine the necessity of including random slopes, we compared a baseline model without random slopes against one with random slopes using the likelihood ratio test. The comparison revealed no significant benefit from adding random slopes (LogLik_1_=6.6, LogLik_2_=8.9; X^2^=4.5, Df=5; p-value=0.474). Consequently, we did not include random slopes in the baseline model.

Stroke outcome (good vs. poor prognosis) was introduced as the independent variable of interest. Additionally, we included an interaction term between stroke outcome and time to test whether patients with different outcomes exhibited distinct trajectories in gene expression over time. Post-hoc analyses were conducted to assess differences between groups at each time point. Marginal means obtained at each time point, along with 95% confidence intervals, are reported. We checked normality of residuals and confirmed the absence of correlation between fitted values and residuals.

### Differential Methylation Regions (DMR) analyses.

For discovering DMRs we used the *comb-p* library[11]. Briefly, this library averages single CpGs *p*-values from adjacent genetic regions after accounting for their correlation. We used the meta-analyzed *p*-values as input and defined a seed *p-*value of 10^-4^, which means that it needs at least one *p*-value ≤ 10^-4^ to extend the region to the next CpG within a 500kb window in our case[12]. We only considered those regions as significant when it had at least 4 CpGs and a Q-value < 0.05 (Sidak correction). DMRs were annotated using GREAT[13].

### Biological pathways analyses

For biological pathways, we used the methylGSA R package using the CpGs’ meta-analyzed *p*-values as input, as we did for DMRs. This library takes as input single CpG *p*-values and conducts a ranked gene set enrichment analysis (GSEA). As one gene might receive annotations from more than one CpG, their significance is averaged using the Robust Rank Aggregation method[14]. Then, three different databases are tested for enrichment: Gene-Ontologies, KEGG and Reactome.

## Data Sharing

Data will be shared upon request of qualified researchers.

## Bioinformatic Resources

Software:

- R-software version 4.2.1.
- Cytoscape version 3.9.1.
- METAL software[15].

| **R-library** | **Version** | **Use** |
| --- | --- | --- |
| Limma | 3.52.4 | Linear models EWAS (DMPs) |
| Ggplot2 | 3.4.1 | Data visualization |
| sva | 3.44.0 | Adjusment batch effect |
| ChAMP | 2.26.0 | Parsing idat files and doing quality controls |
| minfi | 1.42.0 | Parsing idat files and doing quality controls |
| ENmix | 1.32.0 | DMRs analysis |
| MethylGSA | 1.14.0 | Gene-set enrichment analysis |
| coMET | 1.28.0 | Data visualization |
| EpiDISH | 2.16.0 | Differentially methylated cell types |

## Resource table

| **Description** | **Source** | **URL** |
| --- | --- | --- |
| **DNA extraction** - Gentra Puregene Blood kit | Qiagen | https://www.qiagen.com/es-us/products/discovery-and-translational-research/dna-rna-purification/dna-purification/genomic-dna/puregene-kits |
| **DNA extraction** - Autopure LS | Qiagen | https://www.qiagen.com/ch/resources/resourcedetail?id=689194bd-f646-4b8d-a0e9-193f8b6503b7&lang=en |
| **DNA extraction** - Chemagic Magnetic Separation Module I system | Chemagen | https://chemagen.com/products/chemagen-ivd-products/ce-ivd-chemagic-kits/3097-0020-chemagic-dna-plasma200-kit-msm-i/ |
| **DNAm measurement -** Infinium® HumanMethylation450 BeadChip | Illumina | https://www.illumina.com/content/dam/illumina-marketing/documents/products/datasheets/datasheet_humanmethylation450.pdf |
| **DNAm measurement -** Infinium MethylationEPIC v1.0 | Illumina | https://emea.support.illumina.com/downloads/infinium-methylationepic-v1-0-product-files.html |
| **RNA collection -** PAXgene tubes | Qiagen | https://www.qiagen.com/us/products/discovery-and-translational-research/sample-collection-stabilization/rna/paxgene-blood-rna-tubes |
| **RNA extraction -** PAXgene Blood RNA extraction Kit | Qiagen | https://www.qiagen.com/us/products/diagnostics-and-clinical-research/solutions-for-laboratory-developed-tests/paxgene-blood-rna-kit-ivd |
| **Gene expression quantification - GeneChip Human Gene 2.0 ST** | Affymetrix | https://www.thermofisher.com/order/catalog/product/es/en/902113 |

## References methods

1. Mola-Caminal M, Carrera C, Soriano-Tárraga C, Giralt-Steinhauer E, Díaz-Navarro RM, Tur S, et al. PATJ Low Frequency Variants Are Associated With Worse Ischemic Stroke Functional Outcome. Circ Res. 2019;124:114–20.

2. Adams HPJ, Bendixen BH, Kappelle LJ, Biller J, Love BB, Gordon DL, et al. Classification of subtype of acute ischemic stroke. Definitions for use in a multicenter clinical trial. TOAST. Trial of Org 10172 in Acute Stroke Treatment. Stroke. 1993;24:35–41.

3. Aryee MJ, Jaffe AE, Corrada-Bravo H, Ladd-Acosta C, Feinberg AP, Hansen KD, et al. Minfi: a flexible and comprehensive Bioconductor package for the analysis of Infinium DNA methylation microarrays. Bioinformatics. 2014;30:1363–9.

4. Tian Y, Morris TJ, Webster AP, Yang Z, Beck S, Feber A, et al. ChAMP: Updated methylation analysis pipeline for Illumina BeadChips. Bioinformatics. 2017;33:3982–4.

5. Wang Y, Hannon E, Grant OA, Gorrie-Stone TJ, Kumari M, Mill J, et al. DNA methylation-based sex classifier to predict sex and identify sex chromosome aneuploidy. BMC Genomics. 2021;22:1–11.

6. Teschendorff AE, Marabita F, Lechner M, Bartlett T, Tegner J, Gomez-Cabrero D, et al. A beta-mixture quantile normalization method for correcting probe design bias in Illumina Infinium 450 k DNA methylation data. Bioinformatics. 2013;29:189–96.

7. Leek JT, Johnson WE, Parker HS, Fertig EJ, Jaffe AE, Zhang Y, Storey JD TL. sva: Surrogate Variable Analysis. R package version 3420. 2021;

8. Houseman EA, Accomando WP, Koestler DC, Christensen BC, Marsit CJ, Nelson HH, et al. DNA methylation arrays as surrogate measures of cell mixture distribution. BMC Bioinformatics. 2012;13.

9. van Iterson M, van Zwet EW, Heijmans BT, ’t Hoen PAC, van Meurs J, Jansen R, et al. Controlling bias and inflation in epigenome- and transcriptome-wide association studies using the empirical null distribution. Genome Biol. 2017;18.

10. Zheng SC, Breeze CE, Beck S, Teschendorff AE. Identification of differentially methylated cell types in epigenome-wide association studies. Nat Methods. 2018;15:1059–66.

11. Pedersen BS, Schwartz DA, Yang I V., Kechris KJ. Comb-p: Software for combining, analyzing, grouping and correcting spatially correlated P-values. Bioinformatics. 2012;28:2986–8.

12. Smith RG, Pishva E, Shireby G, Smith AR, Roubroeks JAY, Hannon E, et al. A meta-analysis of epigenome-wide association studies in Alzheimer’s disease highlights novel differentially methylated loci across cortex. Nat Commun. 2021;12:3517.

13. McLean CY, Bristor D, Hiller M, Clarke SL, Schaar BT, Lowe CB, et al. GREAT improves functional interpretation of cis-regulatory regions. Nat Biotechnol. 2010;28:495–501.

14. Ren X, Kuan PF. methylGSA: a Bioconductor package and Shiny app for DNA methylation data length bias adjustment in gene set testing. Bioinformatics. 2019;35:1958–9.

15. Willer CJ, Li Y, Abecasis GR. METAL: Fast and efficient meta-analysis of genomewide association scans. Bioinformatics. 2010;26:2190–1.

# Supplemental FIGURES


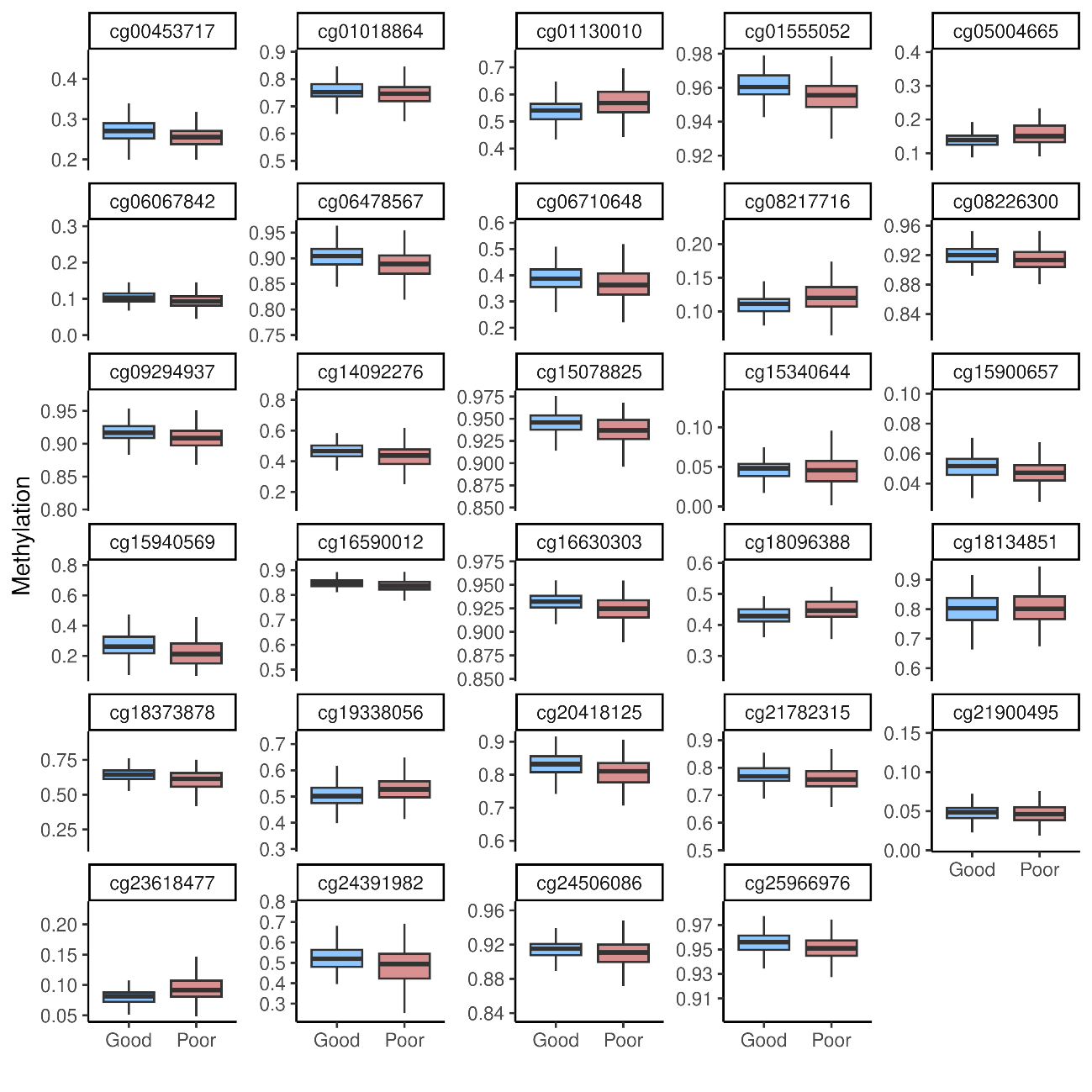


## Supplementary Figure S1.

Boxplots comparing the methylation levels between patients with good and poor prognosis in the subset of CpGs showing a *p*-value < 10^-5^ in the discovery stage.


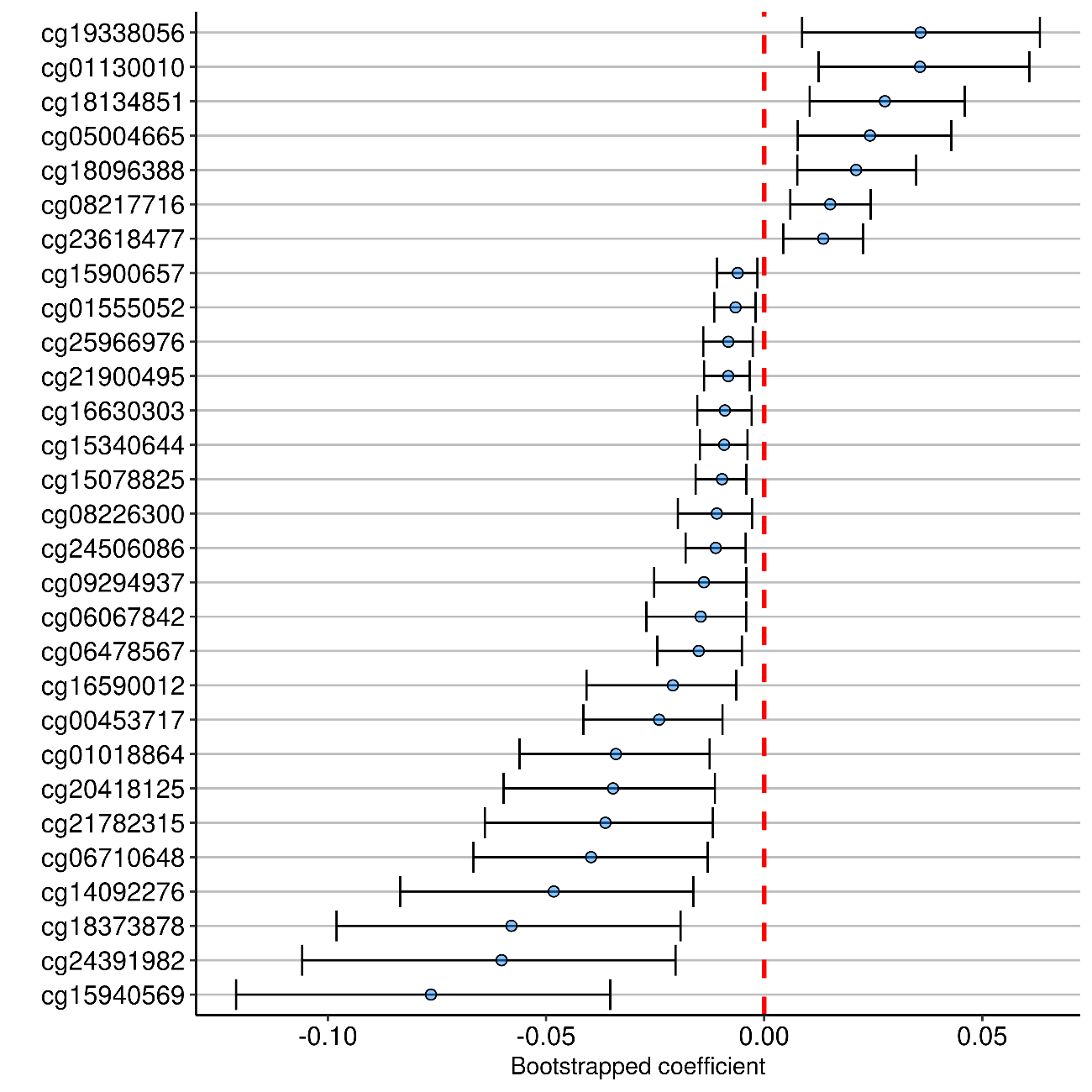


## Supplementary Figure S2.

Bootstrap of those DMPs found in the discovery stage. Each dot corresponds to the original CpG β-coefficient and error bars indicate the bootstrapped 99.9% confidence interval. The vertical red dashed line corresponds to 0 (no effect). Those error bars crossing the vertical red line are not significant after doing the bootstrap. No CpG was lost.


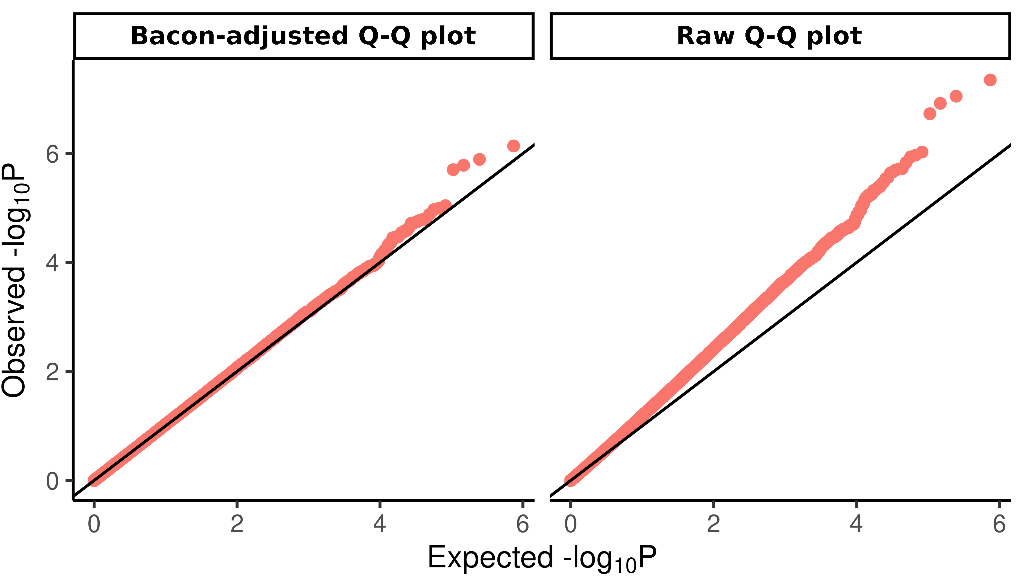


## Supplementary Figure S3.

Q-Q plots showing the *p*-values distribution of the discovery study before and after adjusting for test-statistic inflation with the *Bacon* library.


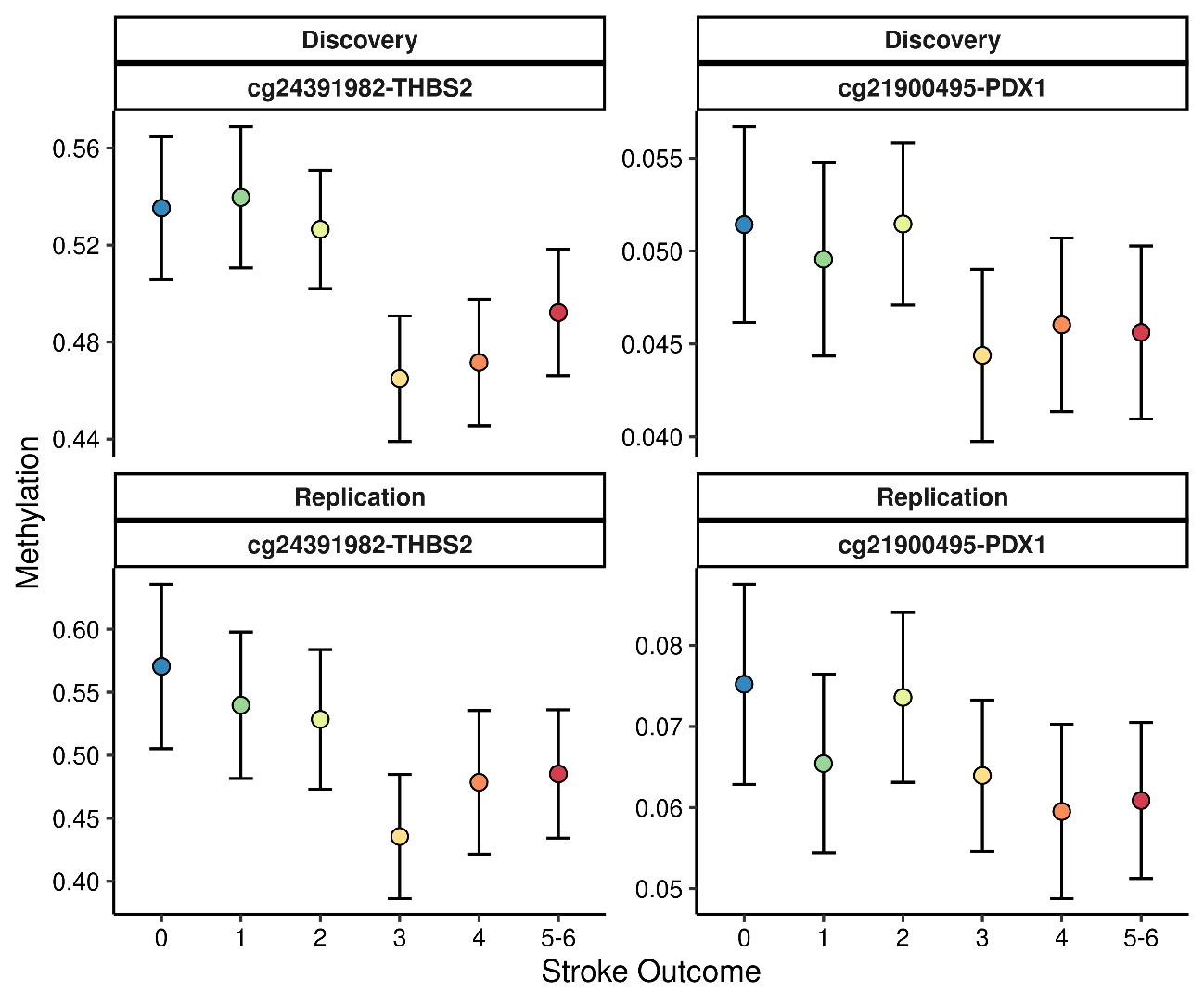


## Supplementary Figure S4.

Methylation levels at THBS2 and PDX1 according to mRS levels. Dots represent marginal means of multivariate models interrogating the association between methylation at these sites and stroke outcome for both discovery and replication stages (see the statistics section). Error bars correspond to 99% confidence interval.


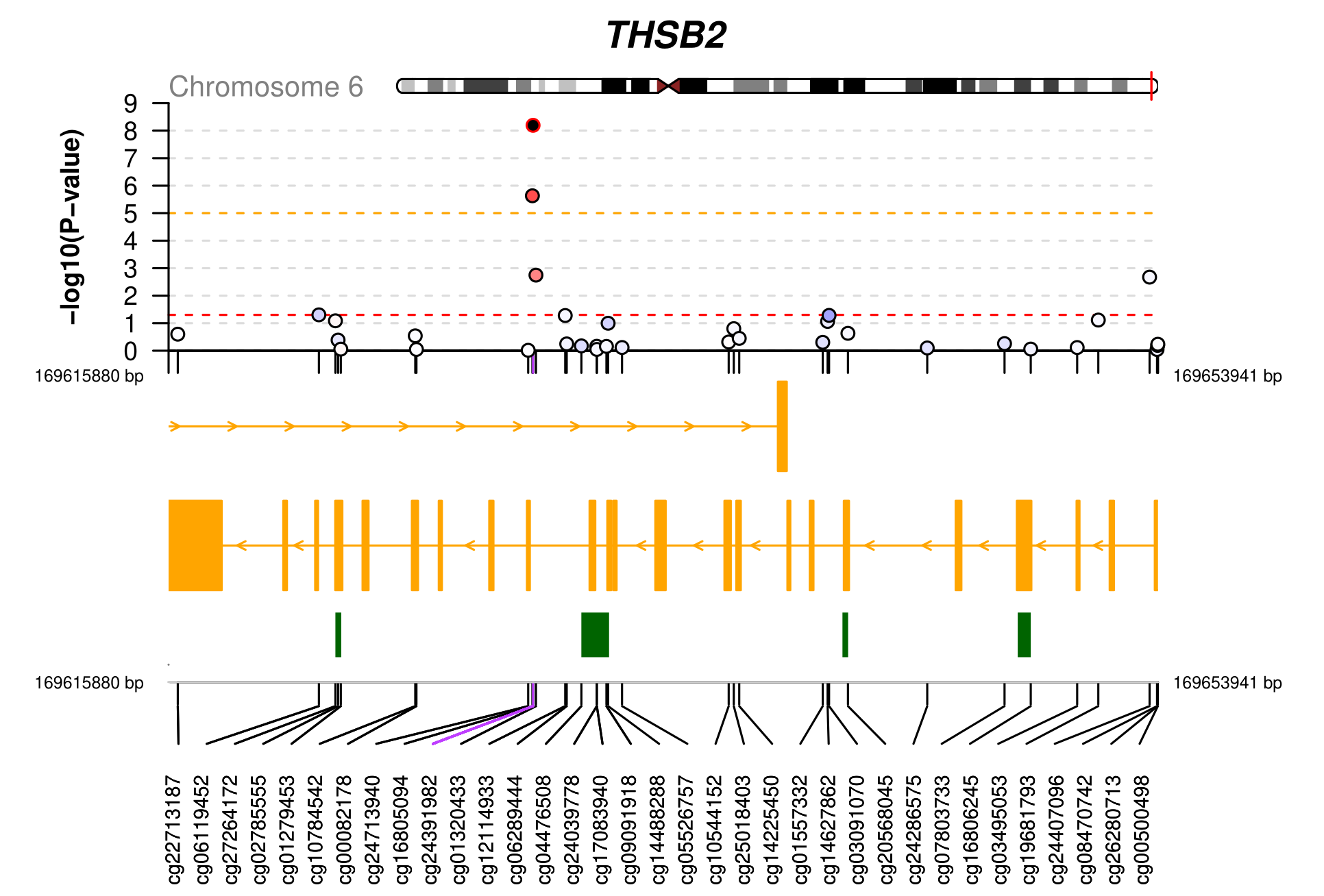


## Supplementary Figure S5.

Locus plot of THBS2 gene. Each dot represents one CpG of THBS2 gene. The X axis corresponds to the gene coordinates, while Y axis shows the -log_10_ *p*-value. These *p*-values correspond to the meta-analyzed results and, as observed, there were two differentially methylated positions with a *p*-value <10^-5^. Gene has been additionally annotated according to exonic regions (yellow boxes) and CpG islands (green boxes).


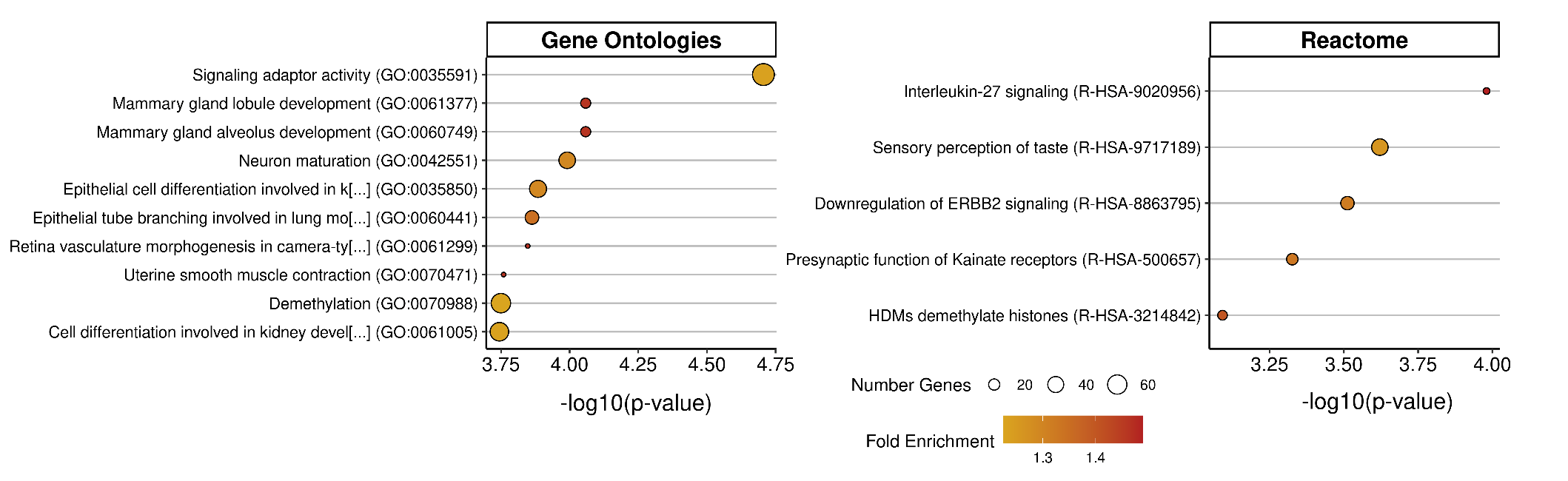


## Supplementary Figure S6.

Gene set enrichment analysis obtained from the meta-analysis. We analyzed the functional enrichment in two different databases: Gene-Ontologies and Reactome. The Y axis corresponds to the top enriched gene-set candidates (Q-value < 0.25). The X axis corresponds to the -log_10_ *p*-value. Color represents the fold enrichment, while dot size to the number of genes included in each gene-set.

# Supplemental tables

| Supplementary Table S1: DNA methylation quality controls in the discovery sample | |
| --- | --- |
| **Technical Run 1, 450K (BasicMar-1)** | |
| *Sample QCs (N=162)* | |
| Sex mismatch | 1 |
| Call rate < 98% | 0 |
| Deviated median intensities | 0 |
| Missing 3 months mRS | 1 |
| Final Sample size | 160 |
| *CpGs QCs (N>450K)* | |
| 1% samples detection *p*-value > .05 | 10438 |
| Beadcount < 3 in 5% samples | 64 |
| Non-CpG probes | 2725 |
| SNP positions | 57595 |
| Multi-hit CpGs | 11 |
| CpGs in X and Y chromosomes | 9607 |
| Final number of CpGs | 405072 |
| **Technical Run 2, 450K (BasicMar-1)** | |
| *Sample QCs (N=90)* | |
| Sex mismatch | 3 |
| Call rate < 98% | 0 |
| Deviated median intensities | 0 |
| Missing 3 months mRS | 1 |
| Final Sample size | 86 |
| *CpGs Qcs (N>450K)* | |
| 1% samples detection *p*-value > .05 | 13332 |
| Beadcount < 3 in 5% samples | 328 |
| Non-CpG probes | 2662 |
| SNP positions | 57018 |
| Multi-hit CpGs | 11 |
| CpGs in X and Y chromosomes | 9515 |
| Final number of CpGs | 402646 |
| **Technical Run 3, EPIC (BasicMar-2)** | |
| *Sample QCs (N=71)* | |
| Sex mismatch | 0 |
| Call rate < 98% | 0 |
| Deviated median intensities | 0 |
| Missing 3 months mRS | 1 |
| Final Sample size | 70 |
| *CpGs Qcs (N>850K)* | |
| 1% samples detection *p*-value > .05 | 20504 |
| Beadcount < 3 in 5% samples | 545 |
| Non-CpG probes | 2836 |
| SNP positions | 94862 |
| Multi-hit CpGs | 11 |
| CpGs in X and Y chromosomes | 16025 |
| Final number of CpGs | 731076 |
| **Merge** | |
| **Final number of samples** | **316** |
| **Common CpGs** | **370344** |

We summarize sample and CpGs quality controls applied to each technical run conforming the discovery sample. Technical runs 1 and 2 conformed the BasicMar-1 cohort, while technical run 3 corresponded to the BasicMar-2 sample. Final number of CpGs after merging the three batches was 370,344 (N=316).

*Keywords*: mRS, modified Rankin scale; SNP, single-nucleotide polymorphism; QC, quality control.

| Supplementary Table S2: Multivariate model showing clinical factors independently associated with stroke outcome | | |
| --- | --- | --- |
| **Variable** | **OR (95% CI)** | ***p*-value** |
| NIHSS 24h | 1.36 (1.27;1.47) | <0.0001 |
| Previous mRS-1 | 2.37 (0.97;5.86) | 0.0594 |
| Previous mRS-2 | 6.30 (2.58;16.6) | <0.0001 |
| Age, year increase | 1.03 (1.00;1.06) | 0.0512 |

This table shows a multivariate logistic regression model interrogating the association between stroke outcome and main clinical variables. Model was built via a forward-stepwise algorithm based on Aikake Information Criterion. The scope model was fully adjusted for all variables: age, sex, hypertension, dyslipidemia, diabetes, atrial fibrillation, smoking status, tissue plasminogen activator treatment, previous mRS and NIHSS at 24h. After several iterations, NIHSS at 24h, previous mRS and age entered into the final model.

*Keywords*: mRS, modified Rankin scale; NIHSS, National Institute of Health Stroke Scale.

|  | Supplementary Table S3: Main Characteristics of the replication sample | | | |
| --- | --- | --- | --- | --- |
|  | **Whole Sample (N=92)** | **Good Outcome (N=37)** | **Poor Outcome (N=55)** | ***p*-value** |
| Age, years | 76.5 [69.0;81.0] | 71.0 [67.0;77.0] | 78.0 [73.0;83.5] | 0.001 |
| Sex, female | 47 (51.1%) | 11 (29.7%) | 36 (65.5%) | 0.002 |
| Hypertension | 54 (58.7%) | 19 (51.4%) | 35 (63.6%) | 0.338 |
| Dyslipidemia | 39 (42.4%) | 17 (45.9%) | 22 (40.0%) | 0.726 |
| Diabetes | 15 (16.3%) | 8 (21.6%) | 7 (12.7%) | 0.398 |
| Smoking | 12 (13.0%) | 5 (13.5%) | 7 (12.7%) | 1.000 |
| NIHSS 24h | 10.5 [5.0;16.2] | 4.0 [2.0;8.0] | 15.0 [8.0;18.0] | <0.001 |
| 3-month mRS |  |  |  | - |
| 0 | 12 (13.0%) | - | - |  |
| 1 | 14 (15.2%) | - | - |  |
| 2 | 11 (12.0%) | - | - |  |
| 3 | 13 (14.1%) | - | - |  |
| 4 | 15 (16.3%) | - | - |  |
| 5 | 8 (8.70%) | - | - |  |
| 6 | 19 (20.7%) | - | - |  |

Values represent frequencies (percentage) or medians (interquartile range) in the whole replication sample and by stroke outcome. Last column shows which variables are significantly associated with stroke outcome and *p*-values have been obtained using the χ^2^ or U Man-Witney tests depending on the type of each predictor variable (categorical or continuous).

*Keywords:* mRS, modified Rankin scale; NIHSS, National Institute of Health Stroke Scale.

|  | Supplementary Table S4: Epigenome-wide association study showing differentially methylated position in patients with poor stroke outcome | | | | | | | | | | | | | |
| --- | --- | --- | --- | --- | --- | --- | --- | --- | --- | --- | --- | --- | --- | --- |
|  | **Annotations** | | | | | **Discovery phase (N=316)** | | | **Replication phase (N=92)** | | | **Meta-Analysis (N=408)** | | |
|  | **CHR** | **BP** | **Gene Illumina** | **Feature** | **Gene Great** | **β** | ***p-*value** | ***p*-value (bacon)** | **β** | ***p-*value** | **Q-value** | ***p-*value** | **Direction** | **Het. *p-*value** |
| cg15940569 | 15 | 27019356 | GABRB3 | TSS1500 | GABRB3 | -0.077 | 9.40E-08 | 7.18E-07 | 0.006 | 8.74E-01 | 8.80E-01 | 3.87E-06 | -+ | 7.34E-03 |
| cg00453717 | 11 | 74344629 | POLD3 | Body | POLD3 | -0.025 | 1.76E-07 | 1.27E-06 | -0.016 | 6.35E-01 | 7.19E-01 | 1.44E-06 | -- | 3.86E-02 |
| cg24506086 | 11 | 12922118 | TEAD1 | Body | TEAD1 | -0.011 | 2.31E-07 | 1.63E-06 | 0.008 | 2.75E-01 | 7.08E-01 | 5.60E-05 | -+ | 6.22E-04 |
| cg08217716 | 10 | 102589250 | PAX2 | 3'UTR | FAM178A | 0.015 | 3.46E-07 | 1.97E-06 | -0.003 | 5.98E-01 | 7.19E-01 | 2.33E-05 | +- | 3.85E-03 |
| **cg24391982** | **6** | **169629902** | **THBS2** | **Body** | **THBS2** | **-0.06** | **1.54E-06** | **8.97E-06** | **-0.078** | **9.17E-04** | **2.66E-02** | **6.39E-09** | **--** | **5.31E-01** |
| cg20418125 | 11 | 133099168 | OPCML | Body | OPCML | -0.035 | 1.73E-06 | 9.97E-06 | 0.023 | 2.88E-01 | 7.08E-01 | 2.16E-04 | -+ | 1.32E-03 |
| cg01018864 | 14 | 96158915 | TCL1B | 3'UTR | TCL1B | -0.034 | 1.86E-06 | 1.06E-05 | 0.019 | 4.55E-01 | 7.08E-01 | 1.24E-04 | -+ | 3.42E-03 |
| cg09294937 | 2 | 219028997 | CXCR1 | Body | CXCR1 | -0.014 | 2.32E-06 | 1.29E-05 | 0.008 | 3.87E-01 | 7.08E-01 | 1.82E-04 | -+ | 2.62E-03 |
| cg15078825 | 1 | 182204191 |  |  | GLUL | -0.01 | 2.95E-06 | 1.60E-05 | 0.011 | 1.29E-01 | 7.08E-01 | 7.05E-04 | -+ | 3.69E-04 |
| cg15340644 | 19 | 13262082 | IER2;STX10 | 5'UTR;TSS1500 | IER2 | -0.009 | 2.99E-06 | 1.62E-05 | -0.011 | 2.52E-01 | 7.08E-01 | 3.23E-06 | -- | 2.24E-01 |
| **cg21900495** | **13** | **28498161** | **PDX1** | **Body** | **PDX1** | **-0.008** | **3.12E-06** | **1.69E-05** | **-0.009** | **4.65E-02** | **6.75E-01** | **4.00E-07** | **--** | **6.39E-01** |
| cg21782315 | 6 | 170531847 |  |  | DLL1 | -0.037 | 3.35E-06 | 1.80E-05 | 0.013 | 6.40E-01 | 7.19E-01 | 1.11E-04 | -+ | 8.69E-03 |
| cg25966976 | 3 | 39170681 | TTC21A | Body | GORASP1 | -0.008 | 3.52E-06 | 1.88E-05 | 0.005 | 2.74E-01 | 7.08E-01 | 3.75E-04 | -+ | 1.52E-03 |
| cg18096388 | 2 | 242800973 | PDCD1 | 1stExon | PDCD1 | 0.021 | 4.17E-06 | 1.87E-05 | 0.021 | 4.64E-01 | 7.08E-01 | 1.10E-05 | ++ | 1.22E-01 |
| cg06478567 | 3 | 183019303 | MCF2L2 | Body | B3GNT5 | -0.015 | 4.37E-06 | 2.28E-05 | 0.007 | 4.56E-01 | 7.08E-01 | 2.29E-04 | -+ | 4.49E-03 |
| cg16630303 | 11 | 118019633 | SCN4B | Body | SCN4B | -0.009 | 5.00E-06 | 2.57E-05 | 0.014 | 7.54E-02 | 7.08E-01 | 1.53E-03 | -+ | 1.87E-04 |
| cg08226300 | 3 | 49695806 | BSN | Body | APEH | -0.011 | 5.43E-06 | 2.76E-05 | 0.008 | 2.64E-01 | 7.08E-01 | 5.25E-04 | -+ | 1.65E-03 |
| cg05004665 | 1 | 20800052 |  |  | CAMK2N1 | 0.024 | 6.01E-06 | 2.59E-05 | 0.017 | 2.38E-01 | 7.08E-01 | 5.54E-06 | ++ | 2.64E-01 |
| cg18373878 | 14 | 101192860 | DLK1 | TSS1500 | DLK1 | -0.058 | 6.12E-06 | 3.07E-05 | 0.016 | 6.57E-01 | 7.19E-01 | 1.66E-04 | -+ | 1.10E-02 |
| cg23618477 | 6 | 169334872 |  |  | THBS2 | 0.014 | 6.61E-06 | 2.82E-05 | -0.017 | 1.38E-01 | 7.08E-01 | 1.13E-03 | +- | 5.59E-04 |
| cg01555052 | 14 | 75098624 |  |  | LTBP2 | -0.007 | 6.89E-06 | 3.41E-05 | 0.005 | 3.86E-01 | 7.08E-01 | 3.97E-04 | -+ | 3.69E-03 |
| cg16590012 | 1 | 1265354 | TAS1R3 | TSS1500 | TAS1R3 | -0.021 | 6.98E-06 | 3.46E-05 | 0.004 | 6.70E-01 | 7.19E-01 | 1.77E-04 | -+ | 1.19E-02 |
| cg18134851 | 14 | 36027972 | RALGAPA1 | Body | INSM2 | 0.028 | 7.99E-06 | 3.34E-05 | 0.016 | 4.35E-01 | 7.08E-01 | 1.72E-05 | ++ | 1.50E-01 |
| cg19338056 | 9 | 14992172 | LOC389705 | TSS1500 | FREM1 | 0.036 | 8.29E-06 | 3.46E-05 | 0.03 | 3.75E-01 | 7.08E-01 | 1.41E-05 | ++ | 1.80E-01 |
| cg15900657 | 10 | 22518317 |  |  | COMMD3 | -0.006 | 8.40E-06 | 4.07E-05 | -0.001 | 8.80E-01 | 8.80E-01 | 6.62E-05 | -- | 4.69E-02 |
| cg01130010 | 3 | 132009122 |  |  | CPNE4 | 0.036 | 8.44E-06 | 3.51E-05 | 0.026 | 4.11E-01 | 7.08E-01 | 1.65E-05 | ++ | 1.62E-01 |
| cg14092276 | 14 | 101192852 | DLK1 | TSS1500 | DLK1 | -0.048 | 9.24E-06 | 4.43E-05 | 0.022 | 5.39E-01 | 7.19E-01 | 3.09E-04 | -+ | 8.03E-03 |
| cg06710648 | 1 | 58716377 | DAB1 | TSS200 | OMA1 | -0.04 | 9.37E-06 | 4.49E-05 | 0.02 | 5.30E-01 | 7.19E-01 | 3.21E-04 | -+ | 7.78E-03 |
| cg06067842 | 10 | 63213385 | TMEM26 | TSS200 | TMEM26 | -0.015 | 9.94E-06 | 4.73E-05 | -0.005 | 6.28E-01 | 7.19E-01 | 3.85E-05 | -- | 9.36E-02 |

Differentially methylated positions (*p*-value < 10^-5^) in patients with poor stroke outcome found in the discovery phase, and validated in the replication stage. All CpG positions have been annotated according to their position, gene (both illumine and GREAT annotations) and their relationship with the gene. We show the β-coefficient and *p*-value for each CpG both in the discovery and replication stages. Besides, in the discovery we show the BACON adjustment, which corrects *p*-values for test-statistic inflation. In the replication, we additionally show the Q-value (false discovery rate). Finally, last three columns show the meta-analysis of both stages (meta-analyzed *p*-value, direction in both studies and heterogeneity *p*-value). Bold CpGs correspond to validated candidates: Nominally significant at both stages and at the meta-analysis. Analyses were adjusted for age, sex, smoking habit, diabetes, hypertension, dyslipidemia, NIHSS at 24 hours and previous mRS.

*Keywords*: BP, base pair; CHR, chromosome; Het, heterogeneity.

| Supplementary Table S5: Differentially methylated cellular types in CpG candidates obtained in the discovery study | | | | | | | |
| --- | --- | --- | --- | --- | --- | --- | --- |
| **CpG** | **Position** | **Gene Illumina** | **Cell Type** | **β (95% CI)** | ***t*** | ***p-*value** | **Q-value** |
| cg15940569 | 15:27019356 | GABRB3 | CD8T | 0.04 (-1.02;1.09) | 0.07 | 9.45E-01 | 9.78E-01 |
|  |  |  | NK | -0.28 (-0.86;0.29) | -0.96 | 3.37E-01 | 8.55E-01 |
|  |  |  | Mono | -0.02 (-0.80;0.77) | -0.05 | 9.64E-01 | 9.80E-01 |
|  |  |  | CD4T | -0.11 (-0.57;0.35) | -0.47 | 6.39E-01 | 8.52E-01 |
|  |  |  | Gran | -0.06 (-0.15;0.03) | -1.37 | 1.73E-01 | 5.02E-01 |
|  |  |  | Bcell | 0.25 (-1.24;1.75) | 0.33 | 7.41E-01 | 9.62E-01 |
| cg00453717 | 11:74344629 | POLD3 | CD8T | 0.1 (-0.23;0.43) | 0.60 | 5.51E-01 | 9.31E-01 |
|  |  |  | NK | 0.00 (-0.18;0.18) | 0.01 | 9.96E-01 | 9.96E-01 |
|  |  |  | CD4T | 0.05 (-0.09;0.20) | 0.73 | 4.67E-01 | 8.52E-01 |
|  |  |  | Gran | -0.02 (-0.05;0.01) | -1.61 | 1.08E-01 | 3.92E-01 |
|  |  |  | Mono | -0.03 (-0.28;0.22) | -0.26 | 7.97E-01 | 9.80E-01 |
|  |  |  | Bcell | -0.17 (-0.65;0.3) | -0.72 | 4.73E-01 | 9.56E-01 |
| cg24506086 | 11:12922118 | TEAD1 | CD8T | -0.12 (-0.33;0.08) | -1.21 | 2.29E-01 | 8.31E-01 |
|  |  |  | Bcell | -0.01 (-0.29;0.28) | -0.05 | 9.57E-01 | 9.91E-01 |
|  |  |  | Gran | 0.00 (-0.02;0.02) | 0.19 | 8.47E-01 | 9.78E-01 |
|  |  |  | NK | -0.02 (-0.13;0.09) | -0.40 | 6.87E-01 | 8.55E-01 |
|  |  |  | Mono | -0.04 (-0.19;0.11) | -0.48 | 6.29E-01 | 9.80E-01 |
|  |  |  | CD4T | -0.02 (-0.11;0.07) | -0.52 | 6.06E-01 | 8.52E-01 |
| cg08217716 | 10:102589250 | PAX2 | Gran | 0.01 (-0.01;0.03) | 0.58 | 5.64E-01 | 9.17E-01 |
|  |  |  | CD4T | 0.01 (-0.10;0.11) | 0.10 | 9.19E-01 | 9.70E-01 |
|  |  |  | CD8T | 0.09 (-0.14;0.32) | 0.78 | 4.35E-01 | 9.31E-01 |
|  |  |  | NK | -0.06 (-0.19;0.06) | -0.95 | 3.41E-01 | 8.55E-01 |
|  |  |  | Mono | 0.11 (-0.06;0.28) | 1.23 | 2.18E-01 | 6.79E-01 |
|  |  |  | Bcell | 0.07 (-0.26;0.40) | 0.41 | 6.84E-01 | 9.62E-01 |
| cg24391982 | 6:169629902 | THBS2 | Bcell | 1.00 (-0.32;2.32) | 1.59 | 1.12E-01 | 8.31E-01 |
|  |  |  | CD8T | -0.04 (-0.97;0.89) | -0.09 | 9.28E-01 | 9.78E-01 |
|  |  |  | NK | -0.10 (-0.61;0.41) | -0.39 | 6.95E-01 | 8.55E-01 |
|  |  |  | Mono | 0.02 (-0.67;0.72) | 0.06 | 9.49E-01 | 9.80E-01 |
|  |  |  | CD4T | -0.41 (-0.81;0.00) | -1.98 | 4.91E-02 | 4.74E-01 |
|  |  |  | Gran | -0.04 (-0.12;0.04) | -0.92 | 3.57E-01 | 7.96E-01 |
| cg20418125 | 11:133099168 | OPCML | Mono | -0.15 (-0.54;0.25) | -0.73 | 4.64E-01 | 8.98E-01 |
|  |  |  | CD8T | 0.07 (-0.46;0.60) | 0.27 | 7.84E-01 | 9.47E-01 |
|  |  |  | NK | 0.13 (-0.16;0.42) | 0.89 | 3.73E-01 | 8.55E-01 |
|  |  |  | CD4T | -0.07 (-0.30;0.16) | -0.59 | 5.53E-01 | 8.52E-01 |
|  |  |  | Gran | -0.03 (-0.07;0.02) | -1.22 | 2.23E-01 | 5.88E-01 |
|  |  |  | Bcell | 0.00 (-0.75;0.75) | 0.01 | 9.92E-01 | 9.92E-01 |
| cg01018864 | 14:96158915 | TCL1B | Mono | -0.05 (-0.45;0.35) | -0.25 | 8.00E-01 | 9.80E-01 |
|  |  |  | CD4T | 0.09 (-0.15;0.32) | 0.73 | 4.66E-01 | 8.52E-01 |
|  |  |  | NK | -0.38 (-0.67;-0.09) | -2.59 | 9.98E-03 | 2.10E-01 |
|  |  |  | Bcell | 0.12 (-0.64;0.87) | 0.31 | 7.60E-01 | 9.62E-01 |
|  |  |  | CD8T | 0.10 (-0.43;0.63) | 0.38 | 7.06E-01 | 9.31E-01 |
|  |  |  | Gran | -0.01 (-0.05;0.04) | -0.38 | 7.06E-01 | 9.56E-01 |
| cg09294937 | 2:219028997 | CXCR1 | Gran | 0.00 (-0.02;0.02) | 0.24 | 8.14E-01 | 9.78E-01 |
|  |  |  | Mono | -0.11 (-0.27;0.06) | -1.28 | 2.00E-01 | 6.79E-01 |
|  |  |  | Bcell | -0.09 (-0.40;0.22) | -0.58 | 5.61E-01 | 9.56E-01 |
|  |  |  | CD4T | 0.01 (-0.08;0.11) | 0.26 | 7.96E-01 | 9.23E-01 |
|  |  |  | CD8T | 0.17 (-0.05;0.39) | 1.50 | 1.36E-01 | 6.55E-01 |
|  |  |  | NK | -0.12 (-0.24;0.00) | -1.91 | 5.68E-02 | 5.49E-01 |
| cg15078825 | 1:182204191 |  | Bcell | 0.11 (-0.17;0.39) | 0.76 | 4.46E-01 | 9.56E-01 |
|  |  |  | NK | -0.09 (-0.20;0.02) | -1.67 | 9.59E-02 | 6.95E-01 |
|  |  |  | CD4T | 0.00 (-0.09;0.09) | 0.00 | 9.97E-01 | 9.97E-01 |
|  |  |  | CD8T | -0.16 (-0.36;0.04) | -1.57 | 1.18E-01 | 6.55E-01 |
|  |  |  | Mono | 0.01 (-0.13;0.16) | 0.17 | 8.63E-01 | 9.80E-01 |
|  |  |  | Gran | -0.01 (-0.03;0.00) | -1.44 | 1.52E-01 | 4.90E-01 |
| cg15340644 | 19:13262082 | IER2;STX10 | Bcell | -0.07 (-0.35;0.21) | -0.47 | 6.41E-01 | 9.62E-01 |
|  |  |  | NK | -0.02 (-0.13;0.09) | -0.36 | 7.23E-01 | 8.55E-01 |
|  |  |  | CD8T | -0.04 (-0.24;0.16) | -0.39 | 6.98E-01 | 9.31E-01 |
|  |  |  | Mono | 0.06 (-0.09;0.20) | 0.73 | 4.64E-01 | 8.98E-01 |
|  |  |  | Gran | 0.00 (-0.02;0.01) | -0.43 | 6.65E-01 | 9.56E-01 |
|  |  |  | CD4T | -0.03 (-0.11;0.06) | -0.61 | 5.44E-01 | 8.52E-01 |
| cg21900495 | 13:28498161 | PDX1 | NK | 0.01 (-0.08;0.10) | 0.30 | 7.66E-01 | 8.55E-01 |
|  |  |  | Mono | 0.03 (-0.09;0.16) | 0.55 | 5.85E-01 | 9.80E-01 |
|  |  |  | Bcell | -0.16 (-0.4;0.08) | -1.34 | 1.82E-01 | 8.31E-01 |
|  |  |  | Gran | 0.00 (-0.02;0.01) | -0.39 | 6.97E-01 | 9.56E-01 |
|  |  |  | CD8T | -0.05 (-0.21;0.12) | -0.53 | 5.98E-01 | 9.31E-01 |
|  |  |  | CD4T | 0.01 (-0.06;0.09) | 0.39 | 6.95E-01 | 8.76E-01 |
| cg21782315 | 6:170531847 |  | CD8T | -0.19 (-0.77;0.40) | -0.62 | 5.35E-01 | 9.31E-01 |
|  |  |  | CD4T | -0.09 (-0.34;0.17) | -0.66 | 5.09E-01 | 8.52E-01 |
|  |  |  | Gran | -0.03 (-0.08;0.02) | -1.11 | 2.69E-01 | 6.50E-01 |
|  |  |  | NK | 0.06 (-0.26;0.38) | 0.39 | 6.94E-01 | 8.55E-01 |
|  |  |  | Bcell | -0.23 (-1.07;0.60) | -0.55 | 5.82E-01 | 9.56E-01 |
|  |  |  | Mono | 0.13 (-0.31;0.57) | 0.59 | 5.57E-01 | 9.80E-01 |
| cg25966976 | 3:39170681 | TTC21A | CD4T | -0.04 (-0.11;0.03) | -1.19 | 2.34E-01 | 8.52E-01 |
|  |  |  | Bcell | 0.1 (-0.12;0.33) | 0.90 | 3.67E-01 | 9.56E-01 |
|  |  |  | Mono | 0.00 (-0.12;0.12) | -0.03 | 9.80E-01 | 9.80E-01 |
|  |  |  | NK | -0.03 (-0.12;0.06) | -0.64 | 5.26E-01 | 8.55E-01 |
|  |  |  | CD8T | -0.07 (-0.23;0.09) | -0.88 | 3.82E-01 | 9.31E-01 |
|  |  |  | Gran | 0.00 (-0.02;0.01) | -0.62 | 5.36E-01 | 9.17E-01 |
| cg18096388 | 2:242800973 | PDCD1 | CD4T | -0.13 (-0.31;0.05) | -1.45 | 1.47E-01 | 8.52E-01 |
|  |  |  | Bcell | -0.05 (-0.64;0.53) | -0.18 | 8.57E-01 | 9.70E-01 |
|  |  |  | Mono | 0.38 (0.07;0.69) | 2.44 | 1.54E-02 | 2.24E-01 |
|  |  |  | NK | 0.14 (-0.08;0.37) | 1.23 | 2.21E-01 | 8.55E-01 |
|  |  |  | CD8T | 0.37 (-0.05;0.78) | 1.74 | 8.27E-02 | 6.55E-01 |
|  |  |  | Gran | -0.01 (-0.05;0.02) | -0.57 | 5.69E-01 | 9.17E-01 |
| cg06478567 | 3:183019303 | MCF2L2 | NK | 0.11 (-0.06;0.28) | 1.23 | 2.21E-01 | 8.55E-01 |
|  |  |  | Gran | -0.03 (-0.06;0.00) | -2.17 | 3.12E-02 | 1.92E-01 |
|  |  |  | Mono | -0.04 (-0.27;0.19) | -0.32 | 7.50E-01 | 9.80E-01 |
|  |  |  | CD4T | 0.02 (-0.11;0.16) | 0.32 | 7.52E-01 | 9.09E-01 |
|  |  |  | CD8T | -0.36 (-0.68;-0.05) | -2.29 | 2.26E-02 | 5.50E-01 |
|  |  |  | Bcell | 0.13 (-0.31;0.57) | 0.58 | 5.64E-01 | 9.56E-01 |
| cg16630303 | 11:118019633 | SCN4B | CD8T | -0.11 (-0.27;0.04) | -1.41 | 1.59E-01 | 6.57E-01 |
|  |  |  | Gran | 0.00 (-0.01;0.02) | 0.31 | 7.58E-01 | 9.56E-01 |
|  |  |  | Bcell | -0.14 (-0.36;0.08) | -1.27 | 2.04E-01 | 8.31E-01 |
|  |  |  | CD4T | -0.01 (-0.07;0.06) | -0.16 | 8.71E-01 | 9.70E-01 |
|  |  |  | NK | -0.02 (-0.1;0.07) | -0.39 | 6.96E-01 | 8.55E-01 |
|  |  |  | Mono | -0.05 (-0.17;0.07) | -0.84 | 4.04E-01 | 8.98E-01 |
| cg08226300 | 3:49695806 | BSN | CD8T | -0.01 (-0.21;0.19) | -0.08 | 9.39E-01 | 9.78E-01 |
|  |  |  | Gran | 0.00 (-0.02;0.02) | -0.03 | 9.74E-01 | 9.82E-01 |
|  |  |  | Bcell | -0.18 (-0.46;0.10) | -1.24 | 2.18E-01 | 8.31E-01 |
|  |  |  | CD4T | -0.02 (-0.11;0.06) | -0.56 | 5.79E-01 | 8.52E-01 |
|  |  |  | NK | -0.05 (-0.16;0.06) | -0.90 | 3.68E-01 | 8.55E-01 |
|  |  |  | Mono | 0.01 (-0.14;0.15) | 0.07 | 9.42E-01 | 9.80E-01 |
| cg05004665 | 1:20800052 |  | CD4T | 0.05 (-0.12;0.22) | 0.57 | 5.71E-01 | 8.52E-01 |
|  |  |  | NK | -0.26 (-0.48;-0.05) | -2.46 | 1.45E-02 | 2.10E-01 |
|  |  |  | Bcell | 0.24 (-0.30;0.79) | 0.88 | 3.82E-01 | 9.56E-01 |
|  |  |  | CD8T | 0.32 (-0.07;0.70) | 1.60 | 1.10E-01 | 6.55E-01 |
|  |  |  | Mono | -0.02 (-0.31;0.27) | -0.13 | 8.98E-01 | 9.80E-01 |
|  |  |  | Gran | 0.03 (0.00;0.06) | 1.85 | 6.54E-02 | 2.71E-01 |
| cg18373878 | 14:101192860 | DLK1 | Mono | 0.66 (-0.06;1.38) | 1.80 | 7.37E-02 | 4.24E-01 |
|  |  |  | Gran | -0.10 (-0.19;-0.02) | -2.49 | 1.33E-02 | 1.29E-01 |
|  |  |  | CD8T | 0.00 (-0.96;0.97) | 0.01 | 9.96E-01 | 9.96E-01 |
|  |  |  | NK | -0.15 (-0.68;0.37) | -0.56 | 5.74E-01 | 8.55E-01 |
|  |  |  | CD4T | -0.25 (-0.67;0.17) | -1.18 | 2.41E-01 | 8.52E-01 |
|  |  |  | Bcell | 0.37 (-0.99;1.74) | 0.54 | 5.93E-01 | 9.56E-01 |
| cg23618477 | 6:169334872 |  | Mono | 0.15 (-0.02;0.32) | 1.71 | 8.77E-02 | 4.24E-01 |
|  |  |  | CD8T | -0.05 (-0.28;0.18) | -0.44 | 6.62E-01 | 9.31E-01 |
|  |  |  | CD4T | 0.04 (-0.06;0.14) | 0.72 | 4.69E-01 | 8.52E-01 |
|  |  |  | Gran | 0.00 (-0.02;0.02) | -0.04 | 9.66E-01 | 9.82E-01 |
|  |  |  | Bcell | 0.21 (-0.12;0.54) | 1.25 | 2.14E-01 | 8.31E-01 |
|  |  |  | NK | -0.03 (-0.15;0.1) | -0.40 | 6.91E-01 | 8.55E-01 |
| cg01555052 | 14:75098624 |  | CD8T | -0.17 (-0.32;-0.01) | -2.09 | 3.79E-02 | 5.50E-01 |
|  |  |  | CD4T | -0.03 (-0.10;0.03) | -1.00 | 3.19E-01 | 8.52E-01 |
|  |  |  | Gran | 0.00 (-0.01;0.01) | -0.02 | 9.82E-01 | 9.82E-01 |
|  |  |  | Mono | -0.05 (-0.16;0.07) | -0.78 | 4.34E-01 | 8.98E-01 |
|  |  |  | Bcell | 0.06 (-0.16;0.28) | 0.54 | 5.88E-01 | 9.56E-01 |
|  |  |  | NK | -0.02 (-0.11;0.07) | -0.47 | 6.38E-01 | 8.55E-01 |
| cg16590012 | 1:1265354 | TAS1R3 | Bcell | -0.05 (-0.55;0.45) | -0.19 | 8.51E-01 | 9.70E-01 |
|  |  |  | Gran | 0.00 (-0.03;0.03) | -0.31 | 7.58E-01 | 9.56E-01 |
|  |  |  | Mono | 0.03 (-0.23;0.29) | 0.22 | 8.25E-01 | 9.80E-01 |
|  |  |  | NK | 0.05 (-0.15;0.24) | 0.46 | 6.43E-01 | 8.55E-01 |
|  |  |  | CD4T | -0.18 (-0.34;-0.03) | -2.34 | 1.97E-02 | 2.86E-01 |
|  |  |  | CD8T | -0.19 (-0.54;0.17) | -1.04 | 2.99E-01 | 9.31E-01 |
| cg18134851 | 14:36027972 | RALGAPA1 | CD8T | -0.22 (-0.83;0.40) | -0.69 | 4.92E-01 | 9.31E-01 |
|  |  |  | Gran | -0.06 (-0.11;0.00) | -2.14 | 3.31E-02 | 1.92E-01 |
|  |  |  | NK | 0.06 (-0.28;0.39) | 0.33 | 7.45E-01 | 8.55E-01 |
|  |  |  | Bcell | 0.07 (-0.80;0.94) | 0.17 | 8.69E-01 | 9.70E-01 |
|  |  |  | Mono | 0.48 (0.03;0.94) | 2.07 | 3.96E-02 | 2.87E-01 |
|  |  |  | CD4T | 0.08 (-0.19;0.35) | 0.59 | 5.56E-01 | 8.52E-01 |
| cg19338056 | 9:14992172 | LOC389705 | NK | -0.08 (-0.41;0.24) | -0.51 | 6.11E-01 | 8.55E-01 |
|  |  |  | CD8T | 0.06 (-0.54;0.65) | 0.20 | 8.46E-01 | 9.78E-01 |
|  |  |  | Mono | 0.29 (-0.16;0.73) | 1.26 | 2.08E-01 | 6.79E-01 |
|  |  |  | Bcell | 0.80 (-0.04;1.65) | 1.87 | 6.26E-02 | 8.31E-01 |
|  |  |  | Gran | 0.00 (-0.05;0.05) | -0.16 | 8.77E-01 | 9.78E-01 |
|  |  |  | CD4T | -0.09 (-0.35;0.17) | -0.66 | 5.08E-01 | 8.52E-01 |
| cg15900657 | 10:22518317 |  | CD8T | -0.04 (-0.14;0.06) | -0.81 | 4.19E-01 | 9.31E-01 |
|  |  |  | Mono | 0.01 (-0.06;0.09) | 0.31 | 7.55E-01 | 9.80E-01 |
|  |  |  | CD4T | 0.00 (-0.04;0.05) | 0.08 | 9.37E-01 | 9.70E-01 |
|  |  |  | Bcell | -0.11 (-0.26;0.03) | -1.49 | 1.37E-01 | 8.31E-01 |
|  |  |  | Gran | 0.00 (-0.01;0.01) | -0.66 | 5.08E-01 | 9.17E-01 |
|  |  |  | NK | -0.01 (-0.06;0.05) | -0.23 | 8.18E-01 | 8.78E-01 |
| cg01130010 | 3:132009122 |  | NK | -0.16 (-0.47;0.14) | -1.05 | 2.97E-01 | 8.55E-01 |
|  |  |  | CD4T | 0.10 (-0.15;0.34) | 0.76 | 4.47E-01 | 8.52E-01 |
|  |  |  | Bcell | -0.12 (-0.92;0.67) | -0.30 | 7.63E-01 | 9.62E-01 |
|  |  |  | Gran | 0.02 (-0.03;0.06) | 0.64 | 5.21E-01 | 9.17E-01 |
|  |  |  | Mono | 0.26 (-0.16;0.67) | 1.19 | 2.34E-01 | 6.79E-01 |
|  |  |  | CD8T | 0.15 (-0.41;0.71) | 0.52 | 6.07E-01 | 9.31E-01 |
| **cg14092276** | **14:101192852** | **DLK1** | Mono | 0.90 (0.29;1.51) | 2.90 | 4.00E-03 | 1.16E-01 |
|  |  |  | CD4T | -0.08 (-0.44;0.27) | -0.46 | 6.47E-01 | 8.52E-01 |
|  |  |  | Bcell | 0.07 (-1.09;1.22) | 0.12 | 9.07E-01 | 9.75E-01 |
|  |  |  | CD8T | 0.12 (-0.70;0.93) | 0.28 | 7.80E-01 | 9.47E-01 |
|  |  |  | NK | -0.10 (-0.54;0.35) | -0.43 | 6.67E-01 | 8.55E-01 |
|  |  |  | **Gran** | **-0.14 (-0.21;-0.07)** | **-3.90** | **1.20E-04** | **1.73E-03** |
| **cg06710648** | **1:58716377** | **DAB1** | NK | 0.02 (-0.36;0.39) | 0.08 | 9.35E-01 | 9.69E-01 |
|  |  |  | CD4T | 0.08 (-0.22;0.38) | 0.53 | 5.99E-01 | 8.52E-01 |
|  |  |  | CD8T | 0.22 (-0.46;0.90) | 0.63 | 5.29E-01 | 9.31E-01 |
|  |  |  | Mono | 0.55 (0.04;1.06) | 2.11 | 3.59E-02 | 2.87E-01 |
|  |  |  | Bcell | 0.27 (-0.7;1.23) | 0.54 | 5.92E-01 | 9.56E-01 |
|  |  |  | **Gran** | **-0.12 (-0.17;-0.06)** | **-3.93** | **1.07E-04** | **1.73E-03** |
| cg06067842 | 10:63213385 | TMEM26 | NK | 0.06 (-0.07;0.19) | 0.92 | 3.60E-01 | 8.55E-01 |
|  |  |  | CD8T | 0.05 (-0.19;0.29) | 0.38 | 7.03E-01 | 9.31E-01 |
|  |  |  | Mono | 0.09 (-0.09;0.27) | 0.98 | 3.27E-01 | 8.62E-01 |
|  |  |  | CD4T | -0.15 (-0.25;-0.04) | -2.75 | 6.35E-03 | 1.84E-01 |
|  |  |  | Bcell | 0.21 (-0.13;0.55) | 1.21 | 2.29E-01 | 8.31E-01 |
|  |  |  | Gran | -0.02 (-0.04;0.00) | -2.05 | 4.11E-02 | 1.98E-01 |

Differentially methylated cell types in the CpG candidates found in the discovery study (10^-5^). Values represent β-coefficients and 95% confidence intervals, t-statistic and p-values for the interaction between stroke outcome and cell fractions. Results have been corrected by multiple testing (false discovery rate). Bolded text indicates a significant interaction at Q-value ≤ 0.05.

*Keywords:* Mono, monocytes; Gran, granulocytes.

| Supplementary Table S6: Interaction between stroke etiology and outcome at 3 months after stroke in the CpG candidates obtained in the discovery study | | | | | |
| --- | --- | --- | --- | --- | --- |
| **CpG** | **Position** | **Gene Illumina** | **F-value** | ***p*-value** | **Q-value** |
| cg15940569 | 15:27019356 | GABRB3 | 0.210 | 8.11E-01 | 8.93E-01 |
| cg00453717 | 11:74344629 | POLD3 | 0.504 | 6.05E-01 | 7.97E-01 |
| cg24506086 | 11:12922118 | TEAD1 | 1.666 | 1.91E-01 | 5.03E-01 |
| cg08217716 | 10:102589250 | PAX2 | 1.159 | 3.15E-01 | 5.78E-01 |
| cg24391982 | 6:169629902 | THBS2 | 1.543 | 2.15E-01 | 5.20E-01 |
| cg20418125 | 11:133099168 | OPCML | 1.945 | 1.45E-01 | 4.20E-01 |
| cg01018864 | 14:96158915 | TCL1B | 1.165 | 3.13E-01 | 5.78E-01 |
| cg09294937 | 2:219028997 | CXCR1 | 4.596 | 1.08E-02 | 1.06E-01 |
| cg15078825 | 1:182204191 |  | 1.130 | 3.24E-01 | 5.78E-01 |
| cg15340644 | 19:13262082 | IER2;STX10 | 0.301 | 7.40E-01 | 8.61E-01 |
| cg21900495 | 13:28498161 | PDX1 | 0.041 | 9.60E-01 | 9.60E-01 |
| cg21782315 | 6:170531847 |  | 1.090 | 3.38E-01 | 5.78E-01 |
| cg25966976 | 3:39170681 | TTC21A | 4.055 | 1.83E-02 | 1.06E-01 |
| cg18096388 | 2:242800973 | PDCD1 | 3.352 | 3.63E-02 | 1.76E-01 |
| cg06478567 | 3:183019303 | MCF2L2 | 1.086 | 3.39E-01 | 5.78E-01 |
| cg16630303 | 11:118019633 | SCN4B | 2.472 | 8.61E-02 | 2.78E-01 |
| cg08226300 | 3:49695806 | BSN | 0.324 | 7.23E-01 | 8.61E-01 |
| cg05004665 | 1:20800052 |  | 0.148 | 8.63E-01 | 8.93E-01 |
| cg18373878 | 14:101192860 | DLK1 | 0.156 | 8.55E-01 | 8.93E-01 |
| cg23618477 | 6:169334872 |  | 4.170 | 1.64E-02 | 1.06E-01 |
| cg01555052 | 14:75098624 |  | 2.733 | 6.66E-02 | 2.42E-01 |
| cg16590012 | 1:1265354 | TAS1R3 | 5.659 | 3.87E-03 | 1.06E-01 |
| cg18134851 | 14:36027972 | RALGAPA1 | 0.882 | 4.15E-01 | 6.68E-01 |
| cg19338056 | 9:14992172 | LOC389705 | 4.454 | 1.24E-02 | 1.06E-01 |
| cg15900657 | 10:22518317 |  | 0.633 | 5.32E-01 | 7.34E-01 |
| cg01130010 | 3:132009122 |  | 0.658 | 5.19E-01 | 7.34E-01 |
| cg14092276 | 14:101192852 | DLK1 | 2.905 | 5.63E-02 | 2.33E-01 |
| cg06710648 | 1:58716377 | DAB1 | 0.298 | 7.42E-01 | 8.61E-01 |
| cg06067842 | 10:63213385 | TMEM26 | 0.820 | 4.41E-01 | 6.74E-01 |

Moderation effect of stroke etiology on the relationship between DNA methylation and stroke outcome in the CpG candidates found in the discovery study (10^-5^). Values represent F-statistic and *p*-values for the interaction between stroke outcome and stroke etiology. Results have been corrected by multiple testing (false discovery rate).

|  | Supplementary Table S7: Differentially methylated positions associated with stroke outcome in the Meta-Analysis Study | | | | | | | |
| --- | --- | --- | --- | --- | --- | --- | --- | --- |
|  | **CHR** | **BP** | **Gene Illumina** | **Feature** | **Gene GREAT** | ***p-*value** | **Direction** | **Het. *p-*value** |
| cg24391982 | 6 | 169629902 | THBS2 | Body | THBS2 | 6.39E-09 | -- | 0.5285 |
| cg21900495 | 13 | 28498161 | PDX1 | Body | PDX1 | 4.00E-07 | -- | 0.6665 |
| cg22520791 | 5 | 178784097 |  |  | ADAMTS2 | 1.38E-06 | -- | 0.3294 |
| cg00453717 | 11 | 74344629 | POLD3 | Body | POLD3 | 1.44E-06 | -- | 0.03858 |
| cg16805094 | 6 | 169629875 | THBS2 | Body | THBS2 | 2.33E-06 | -- | 0.9296 |
| cg15340644 | 19 | 13262082 | IER2;STX10 | 5'UTR;TSS1500 | IER2 | 3.23E-06 | -- | 0.2255 |
| cg15940569 | 15 | 27019356 | GABRB3 | TSS1500 | GABRB3 | 3.87E-06 | -+ | 0.00734 |
| cg24414363 | 22 | 42336273 | CENPM | TSS200;Body | CAMK4N22 | 4.89E-06 | ++ | 0.7258 |
| cg07323648 | 17 | 35291127 |  |  | LHX1 | 5.20E-06 | -- | 0.5418 |
| cg05004665 | 1 | 20800052 |  |  | CAMK2N1 | 5.54E-06 | ++ | 0.2649 |

This table shows the CpG candidates nominally associated (*p-*value10^-5^) with stroke outcome in the meta-analysis study. Differentially methylated positions were annotated according to their position, annotated gene (both illumine and GREAT annotations) and relationship with the gene. Meta-analysis was obtained considering all the CpGs obtained in both cohorts (discovery and replication) after applying quality controls (370,344 in the discovery and 358,834 in the replication sample). For each candidate we show the meta-analyzed *p­*-value, the direction in both studies and the heterogeneity *p*-value.

*Keywords:* BP, base pair; CHR, chromosome; Het, heterogeneity.

| Supplementary Table S8: Main characteristics of the sample with gene expression data (N=18) | |
| --- | --- |
| Age, years | 78.0 (72.8;83.0) |
| Sex, female | 8 (44.4%) |
| Hypertension | 16 (88.9%) |
| Dyslipidemia | 6 (33.3%) |
| Diabetes | 6 (33.3%) |
| Atrial Fibrillation | 11 (61.1%) |
| Smoking | 5 (27.8%) |
| TOAST |  |
| Atherothrombotic | 2 (11.1%) |
| Cardioembolic | 13 (72.2%) |
| Undetermined | 3 (16.7%) |
| Baseline NIHSS | 13.5 (6.50;18.0) |
| NIHSS 24h | 3.50 (0.25;8.00) |
| rTPA treatment | 14 (77.8%) |
| Previous mRS |  |
| 0 | 14 (77.8%) |
| 1 | 3 (16.7%) |
| 2 | 1 (5.56%) |
| 3-month mRS |  |
| 0 | 4 (22.2%) |
| 1 | 6 (33.3%) |
| 2 | 3 (16.7%) |
| 3 | 2 (11.1%) |
| 4 | 2 (11.1%) |
| 5 | 0 (0.0%) |
| 6 | 1 (5.56%) |

Values represent frequencies (percentage) or medians (interquartile range).

*Keywords*: mRS, modified Rankin scale; NIHSS, National Institute of Health Stroke Scale; TPA, tissue plaminogen activator.

| Supplementary Table S9: Differentially Methylated regions in patients with poor outcome | | | | | | |
| --- | --- | --- | --- | --- | --- | --- |
| **CHR** | **Start** | **End** | **Gene GREAT** | ***p*-value** | **Q-value** | **Nº CpGs** |
| 6 | 29648160 | 29648756 | ZFP57 | 2.03E-14 | 1.30E-11 | 19 |
| 17 | 6899084 | 6899577 | ALOX12 | 2.16E-12 | 1.67E-09 | 11 |
| 17 | 47287409 | 47287577 | ABI3 | 3.04E-10 | 6.87E-07 | 6 |
| 2 | 3704500 | 3704773 | ALLC | 3.27E-10 | 4.55E-07 | 5 |
| 7 | 27183989 | 27184188 | HOXA5 | 2.77E-05 | 5.15E-02 | 8 |

Significant differentially methylated regions associated with stroke outcome. These regions have been obtained using as input the meta-analysis results. For each region we show the chromosome, the starting base-pair and the last one, as well as GREAT annotations. The significance was tested using the *comb-p* library and we report the raw *p*-value, the Q-value (Sidak adjustment) and the number of CpGs conforming the region. We only considered DMRs conformed by at least 4 CpGs.
